# Genetic characterization of 2 *Ceutorhynchus* (Coleoptera: Curculionidae) weevils with mitogenomes and insights into the phylogeny and evolution of related weevils

**DOI:** 10.1093/jisesa/ieae038

**Published:** 2024-03-27

**Authors:** Xinghao Li, Rufan Li, Fuqiang Rao, Rong An, Jianchang Li, Zhenlan Zhang, Yonghong Li, Deguang Liu

**Affiliations:** Key Laboratory of Plant Protection Resources and Pest Management of Ministry of Education, College of Plant Protection, Northwest A&F University, Yangling, Shaanxi 712100, China; Key Laboratory of Plant Protection Resources and Pest Management of Ministry of Education, College of Plant Protection, Northwest A&F University, Yangling, Shaanxi 712100, China; Key Laboratory of Plant Protection Resources and Pest Management of Ministry of Education, College of Plant Protection, Northwest A&F University, Yangling, Shaanxi 712100, China; Hybrid Rapeseed Research Center of Shaanxi Province, Yangling, Shaanxi 712100, China; Hybrid Rapeseed Research Center of Shaanxi Province, Yangling, Shaanxi 712100, China; Hybrid Rapeseed Research Center of Shaanxi Province, Yangling, Shaanxi 712100, China; Hybrid Rapeseed Research Center of Shaanxi Province, Yangling, Shaanxi 712100, China; Key Laboratory of Plant Protection Resources and Pest Management of Ministry of Education, College of Plant Protection, Northwest A&F University, Yangling, Shaanxi 712100, China; Key Laboratory of Integrated Pest Management on the Loess Plateau of Ministry of Agriculture and Rural Affairs, College of Plant Protection, Northwest A&F University, Yangling, Shaanxi 712100, China

**Keywords:** mitochondrial genome, DNA barcoding, Curculionoidea, phylogenetics, ecological niche specialization

## Abstract

The rape stem weevil (*Ceutorhynchus asper* Roel.) and its close relatives primarily breed on cruciferous plants and cause severe damage to rapeseed production. However, their genetic and molecular information is still scarce. Here, we generated mitogenomes for both *C. asper* and *Ceutorhynchus albosuturalis.* The lengths of the 2 mitochondrial genomes are 14,207 bp (*C. asper*) and 15,373 bp (*C. albosuturalis*), and both weevils exhibit identical numbers of protein-coding genes with the absence of trnI. A + T contents for both mitogenomes are high (80% and 79.9%, respectively). Haplotype and genetic distance analyses showed that the genetic differentiation of *C. asper* populations in northwestern China is low. Based on 5 datasets from mitogenomes, phylogenetic analyses with maximum-likelihood and Bayesian methods show that both species (*C. asper* and *C. albosuturalis*) fall in the CCCMS clade (Curculioninae, Conoderinae, Cossoninae, Molytinae, and Scolytinae) of Curculionidae and belong to clades H and I of the genus *Ceutorhynchus*, respectively. Larvae of the clade H weevils mainly are borers in petioles or stems of cruciferous plants, while larvae of the clade I weevils mainly inhabit the fruits of the same plants, suggesting that ecological niche specialization can play a critical role in the diversification of *Ceutorhynchus* species. This study generates baseline molecular and genetic information for future research of *Ceutorhynchus*-related taxa and provides insights into the phylogeny and evolution of Curculionidae.

## Introduction

Weevils are one of the most important groups of insect pests that occur on the oilseed rape (*Brassica napus* L.). *Ceutorhynchus napi*, *Ceutorhynchus rapae*, and *Ceutorhynchus asper* are currently among the most detrimental weevil species on the oilseed rape; among these, *C. napi* is predominantly distributed in Europe, and *C. asper* is mainly found in northwestern China (Büechi 1988, Li et al. 2009, Williams 2010, Sandrine and Peter 2017, Schaefer et al. 2017, Lu et al. 2019). The 3 species share a similar life cycle and feed on the same cruciferous plants. The resulting symptoms of their feeding on the host plants are comparable, as females deposit eggs on developing stems, which will twist, split, and form an S-shape, causing plant deformation and significant yield losses (Büechi 1988, Li et al. 2009, Williams 2010, Schaefer et al. 2017).

All the above-mentioned 3 species belong to the family Curculionidae of the superfamily Curculionoidea. Curculionoidea is unequivocally identified as a monophyletic group and encompasses approximately 62,000 species distributed among 5,800 described genera, rendering them one of the most diverse groups of Coleoptera (Oberprieler et al. 2007, McKenna et al. 2009). Curculionidae is the largest family of Curculionoidea, comprised of approximately 4,600 genera and 51,000 species globally (Oberprieler 2014). The diversification of weevils is believed to be linked to the radiation of angiosperms, particularly eukaryotic dicotyledonous plants (McKenna et al. 2009, Letsch et al. 2018).

Due to the high diversity of weevils, their phylogenetic relationships remain incompletely resolved. The Curculionoidea were earlier variously classified as many different families and subfamilies until Kuschel (1995) introduced a “phylogenetic” classification that recognized only 6 families, Nemonychidae, Anthribidae, Belidae, Attelabidae, Brentidae, and Curculionidae. Subsequently, this superfamily was expanded by Oberprieler et al. (2007) and Shin et al. (2018) to comprise 8 families (adding Caridae and Cimberididae), and a ninth, extinct family, Mesophyletidae, was recently recognized by Clarke et al. (2018). Although the monophyletic status of each of these families is well supported, the phylogenetic relationships among subfamilies of Curculionidae remain uncertain (Marvaldi et al. 2002, Gunter et al. 2016, Clarke et al. 2018, Shin et al. 2018, Song et al. 2020, Smit et al. 2022, Haran et al. 2023, Hsiao et al. 2023).

In addition, the biology of *Ceutorhynchus* and allied genera remains poorly understood despite previous studies indicating their preference for cruciferous plants and other potential hosts (Gillett et al. 2014, D’Ottavio et al. 2023, Daum et al. 2023, Katovich et al. 2023). This brings a huge challenge for the taxonomic classification of these species, as there are only a limited number of defining characteristics for many closely related species, especially of the large genus *Ceutorhynchus*. Moreover, despite the considerable interest in Curculionoidea, there remains a dearth of knowledge regarding phylogenetic relationships among the species, including those of the genus *Ceutorhynchus*. Thus, we sequenced the mitochondrial genomes of 2 weevil species, namely *C. asper* and *C. albosuturalis*. *Cox*1 sequencing was also performed on these rapeseed weevils collected from various places to assess their phylogenetic positions in the genus and their intraspecific genetic diversity. Both DNA barcoding and mitochondrial genome sequencing were employed to address the following issues: (i) resolving separate species issues of *C. napi*, *C. rapae*, and *C. asper*; (ii) examining the phylogenetic relationships of these species and determining the phylogenetic positions of both *C. asper* and *C. albosuturalis*; and (iii) assessing phylogenetic relationships in and among different weevil clades in Curculionoidea and the evolutionary implications.

## Materials and Methods

### Specimen Collection and Morphological Identification

Adult specimens of *C. albosuturalis* were collected in Yangling (34.15° N, 108.06.15° E) of Shaanxi Province in 2021. Samples of *C. asper* were collected at several locations in Shaanxi and Gansu provinces (Supplementary Table S1). The specimens of *C. asper* were initially identified based on morphological characteristics (Li et al. 2009). Representative specimens were deposited in the Entomological Museum of Northwest A&F University, Xianyang, China. Genetic identification was conducted by comparison with *cox1* sequences of target species in the databases of BOLD (Barcode of Life Database: http://www.boldsystems.org - Identification section) and NCBI GenBank. All 93 specimens were preserved in 95% ethanol at −20 °C, and total DNA was extracted from muscle tissues according to the user manual of the DNeasy Blood & Tissue kit (QIAGEN, Beijing, China). High-throughput sequencing for subsequent recovery of mitochondrial genomes was conducted on the Illumina Novaseq 6000 platform (Illumina, Alameda, California) at Novogene Company in Beijing, using the 150-bp paired-end sequencing technique. Raw data were processed to remove adapters, and low-quality reads (containing ambiguous bases or shorter than 88 bp) were removed by using the program Fastp (Chen et al. 2018). After removing low-quality sequences, de novo assembly was performed with the Getorganelle-1.7.6.1 software (Jin et al. 2020). The 2 mitochondrial genomes were submitted to GenBank with the accession numbers OR255927 and OR255928, respectively.

### Sequence Annotation and Analyses

Mitochondrial genome annotation was conducted online using the MITOS2 web server (available at http://mitos2.bioinf.uni-leipzig.de/index.py). Maps of the mitochondrial genomes were plotted by using the visualization tool Organellar Genome DRAW (https://chlorobox.mpimp-golm.mpg.de/OGDraw.html) (Greiner et al. 2019). Base composition, AT- and GC-skew, codon usage, and relative synonymous codon usage (RSCU) were determined by using PhyloSuite v2.53.5 (Zhang et al. 2020). DnaSP v01.54.200 was used to compute nucleotide diversity (Pi) through sliding-window analysis (window size of 200 bp and step size of 20 bp), as well as the nonsynonymous/synonymous substitution rates (*K*_a_/*K*_s_) for the 13 protein-coding genes (PCGs) (Rozas et al. 2017). Genetic differentiation (i.e., genetic distances) between taxa was estimated by using the Kimura 2-parameter (K2P) model in MEGA X (Kumar et al. 2018). The substitution saturation of PCGs per codon position was assessed via the Xia test implemented in the DAMBE program v4.81.57 (Xia and Xie 2001).

### Barcoding DNA Analyses

Thirty-six specimens of *C. asper* were sequenced for the standard *cox1* barcoding region using the universal primer pair LCO1490/HCO2198 (Supplementary Table S1) (Folmer et al. 1994). In addition, the *cox1* sequences of *C. napi* (5 samples) and *C. rapae* (7 samples) were downloaded from NCBI GenBank, sequence alignment was performed with the ClustalW algorithm in MEGA X, and pairwise-genetic distances were calculated with the K2P model (Supplementary Table S2) (Kumar et al. 2018). Haplotype diversity was calculated by using the software DnaSP (Rozas et al. 2017). Arlequin v3.5 was used to conduct analyses of analysis of molecular variance (AMOVA) (Excoffier and Lischer 2010). For maximum-likelihood (ML) analyses, the command “‐spp” was used to allow each partition to have its own evolution rate, and a 1,000-replicate bootstrapping was performed by using the “ultrafast” option implemented in IQ-TREE (Minh et al. 2020). The ModelFinder program (“MFP”) was used to determine substitution models (Kalyaanamoorthy et al. 2017). The *cox1* haplotype median-joining network was constructed using the software Network by employing default parameters (Bandelt et al. 1999). The newly generated barcoding DNA sequences have been deposited in GenBank with accession numbers OR227438–OR227473.

### Phylogenetic Inference and Ancestral Character State Reconstruction

A total of 107 mitochondrial genomes were used to represent 5 families and 17 subfamilies of Curculionoidea. Three species of Chrysomelidae were selected as outgroups (i.e., *Acanthoscelides obtectus*, *Callosobruchus maculatus*, and *Psylliodes chrysocephala*) (Supplementary Table S3). Additionally, in a second analysis, we used 89 *cox1* sequences to assess the specific phylogenetic placement of both *C. asper* and *C. albosuturalis* in the genus *Ceutorhynchus* (Supplementary Table S2).

To evaluate the phylogenetic relationships among species of Curculionoidea and accurately determine the placement of *C. asper* and *C. albosuturalis*, a variety of analytical methods for phylogenetic inference were employed. Specifically, we employed Bayesian inference and ML analyses to perform phylogenetic inferences on 5 datasets: (i) PCG (all codon positions of PCGs); (ii) PCG12 (first and second codon positions of PCGs); (iii) ALL (comprising all 37 mitochondrial genes); (iv) PCGAA (13 amino acid sequences); (v) SRH (the sequence of SRH [stationary, reversible, and homogeneous] model violations after filtering with IQ-TREE v2.0-rc1) (Naser-Khdour et al. 2019, Minh et al. 2020). Phylogenetic trees were inferred with the partitioned-ML and -heterotachtic model (General Heterogeneous evolution On a Single Topology, GHOST) (Crotty et al. 2020). For the PCGAA matrix, the posterior mean site frequency (PMSF) method was used in IQ-TREE (Wang et al. 2018). The Bayesian inference for all matrices was conducted in MrBayes using the PhyloSuite software (Huelsenbeck and Ronquist 2001, Zhang et al. 2020).

The ancestral state of larval feeding was reconstructed from a *cox1* dataset containing 89 *Ceutorhynchus* species by using the ML method in Mesquite v2.75 (http://mesquiteproject.org). The likelihood-based method has been considered a good approach for reconstructing ancestral states (Pagel 1999). For this analysis, the “Markov k-state 1 parameter model” (for which “forward” and “backward” transition rates are assumed to be equal) was used. Sources of data for the larval feeding character are provided in Supplementary Table S11.

## Results

### Genome Organization and Nucleotide Composition

**Fig. 1.**
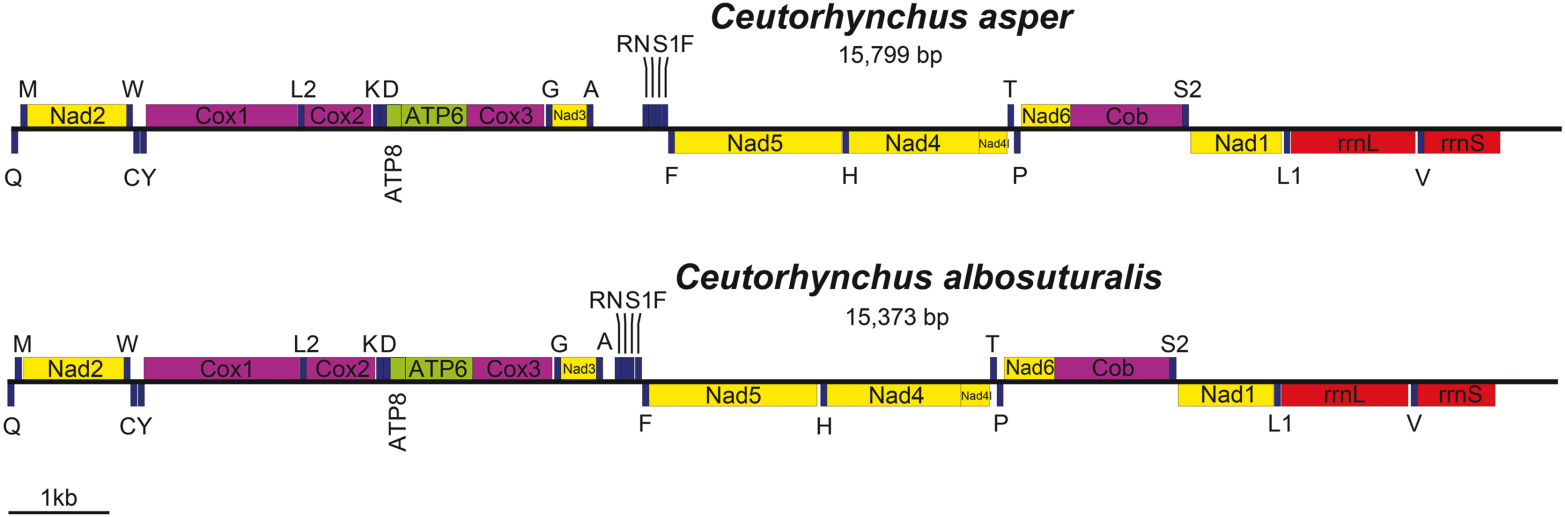
Mitochondrial genome organization of 2 *Ceutorhynchus* weevils: A) *C. asper* and B) *C. albosuturalis*. Genes labeled above the line are transcribed in the direction from left to right, while genes labeled below the line are transcribed from right to left. Blue boxes represent transfer RNAs; yellow boxes represent NADH dehydrogenase subunits; purple boxes represent cytochrome c oxidase subunits and cytochrome b; green boxes represent ATP synthase subunits; red boxes represent ribosomal RNAs.

Our sequencing for *C. asper* and *C. albosuturalis* produced raw paired-end reads of 21434592 and 20739487, clean reads of 20142081 and 19620078, and assembled reads of 18118 and 26498, respectively. The average coverages of the mitochondrial genomes for both species were 378x and 276x, respectively. The assembled mitogenomes of both species had a length of 14,207 bp (*C. asper*) and 15,373 bp (*C. albosuturalis*), respectively. Both species exhibited identical numbers of PCGs and rRNA and tRNA arrangements in mitogenomes. However, the 2 mitochondrial genomes are incomplete, and *trnI* was found to be absent in both species (Table 1; Fig. 1). The mitochondrial genomes of the 2 species are compact, with 30 intergenic regions in *C. asper* and 31 intergenic regions in *C. albosuturalis* and the largest intergenic region located between tRNA^Ala^ and tRNA^Arg^ (*C. asper*: 501 bp, *C. albosuturalis*: 118 bp). Both species have PCG translation initiators of 3 codons (ATT, ATG, ATA) and terminators of TAA and TAG. For both, nad4 has TAA as the terminator.

**Table 1.** Molecular characters of mitogenomes for *Ceutorhynchus asper* and *Ceutorhynchus albosuturalis*

Gene/region	Ceutorhynchus asper							Ceutorhynchus albosuturalis						
	Code	Position	Strand	Size (bp)	Start codon	Stop codon	IGN	Code	Position	Strand	Size (bp)	Start codon	Stop codon	IGN
tRNA^Gln^	Q	1–69	N	69			4	Q	1–69	N	69			24
tRNA^Met^	M	74–143	J	70			15	M	94–162	J	69			0
ND2	–	159–1,157	J	998	ATG	TAA	−2	–	163–1,176	J	1,014	ATT	TAA	−2
tRNA^Trp^	W	1,156–1,219	J	64			1	W	1,175–1,238	J	64			6
tRNA^Cys^	C	1,221–1,286	N	66			7	C	1,245–1,309	N	65			4
tRNA-^Tyr^	Y	1,294–1,360	N	66			−8	Y	1,314–1,380	N	67			−8
COI	–	1,353–2,897	J	1,545	ATT	TAA	2	–	1,373–2,917	J	1,545	ATT	TAA	3
tRNA^Leu^	L2	2,900–2,964	J	65			0	L2	2,921–2,985	J	65			0
COII	–	2,965–3,645	J	681	ATT	TAA	12	–	2,986–3,666	J	681	ATA	TAA	20
tRNA^Lys^	K	3,658–3,728	J	71			−1	K	3,687–3,757	J	71			−1
tRNA^Asp^	D	3,728–3,793	J	66			0	D	3,757–3,821	J	65			0
ATP8	–	3,794–3,949	J	156	ATT	TAA	−7	–	3,822–3,977	J	156	ATT	TAA	−7
ATP6	–	3,943–4,614	J	672	ATG	TAA	−1	–	3,971–4,642	J	672	ATG	TAA	−1
COIII	–	4,614–5,402	J	789	ATG	TAA	20	–	4,642–5,430	J	789	ATG	TAA	18
tRNA^Gly^	G	5,423–5,486	J	64			0	G	5,449–5,512	J	64			0
ND3	–	5,487–5,840	J	354	ATA	TAG	−2	–	5,513–5,866	J	354	ATA	TAG	−2
tRNA^Ala^	A	5,839–5,904	J	66			118	A	5,865–5,931	J	67			501
tRNA^Arg^	R	6,023–6,085	J	63			−1	R	6,433–6,496	J	64			−1
tRNA^Asn^	N	6,085–6,149	J	65			0	N	6,496–6,561	J	66			0
tRNA^Ser^	S1	6,150–6,216	J	67			7	S1	6,562–6,628	J	67			1
tRNA^Glu^	E	6,224–6,290	J	67			4	E	6,630–6,692	J	63			2
tRNA^Phe^	F	6,295–6,359	N	65			0	F	6,695–6,758	N	64			0
ND5	–	6,360–8,028	N	1,669	ATA	TAA	33	–	6,759–8,463	N	1,705	ATT	TAG	6
tRNA^His^	H	8,062–8,124	N	63			0	H	8,470–8,534	N	65			−3
ND4	–	8,125–9,454	N	1,330	ATG	T	−7	–	8,532–9,864	N	1,333	ATG	T	−7
ND4L	–	9,448–9,738	N	291	ATG	TAA	5	–	9,858–10,148	N	291	ATG	TAA	4
tRNA^Thr^	T	9,744–9,810	J	67			0	T	10,153–10,217	J	65			0
tRNA^Pro^	P	9,811–9,874	N	64			8	P	10,218–10,284	N	67			8
ND6	–	9,883–10,383	J	501	ATT	TAA	0	–	10,293–10,793	J	501	ATT	TAA	1
CYTB	–	10,384–11,523	J	1,140	ATG	TAA	−1	–	10,795–11,934	J	1,140	ATG	TAG	−2
tRNA^Ser^	S2	11,523–11,590	J	68			17	S2	11,933–11,999	J	67			17
ND1	–	11,608–12,555	N	948	ATA	TAG	4	–	12,017–12,943	N	927	ATA	TAG	25
tRNA^Leu^	L1	12,560–12,625	N	66			4	L1	12,969–13,031	N	63			7
16SrRA	–	12,630–13,888	N	1,259			27	–	13,039–14,307	N	1,269			24
tRNA^Val^	V	13,916–13,978	N	63			−1	V	14,332–14,396	N	65			−1
12SrRA	–	13,978–14,752	N	775			0	–	14,396–15,172	N	777			0
AT-rich region	–	14,753–15,373	–	620				–	15,173–15,799	–	627			

IGN shows intergenic regions (positive values) and overlaps (negative values).

The A + T content for the total mitogenomes of the 2 species was high (*C. albosuturalis*: 80%, *C. asper*: 79.9%). For the genes of the mitogenomes, the highest A + T content was found in *atp8* (*C. albosuturalis*: 85.9 %, *C. asper*: 83.3%), and the lowest for *cox1* (*C. albosuturalis*: 69.1%, *C. asper*: 68.4%). The G + C content at all codon positions in *C. albosuturalis* and *C. asper* PCGs are 22.6% and 24.5%, respectively; the G + C content in the second codon position was 29.6% and 29.9%, and 10.9%, and 15.2%, respectively (Supplementary Table S4). AT skews of N-strand (*C. albosuturalis*: −0.16, *C. asper*: −0.14) and J-strand (*C. albosuturalis*: −0.05, *C. asper*: −0.04) and GC-skew in the N-strand’s GC skew (*C. albosuturalis*: 0.18, *C. asper*: 0.24) indicated a relatively higher proportion of G over C on the N-strand, but the opposite was true for the J-strand (*C. albosuturalis*: −0.12, *C. asper*: −0.11) (Table 1; Supplementary Fig. S1).

In the mitogenomes of species in Curculionoidea, the total A + T content ranges from 63.7% to 78.8%. In specific regions, the A + T content varies between 69% and 83.5% for rRNA and from 68.5% and 81.2% for tRNA (Supplementary Table S4; Supplementary Fig. S1A). *Cox1* has the lowest A + T content (67.1%) among all PCGs, followed by *cox3* (69.5%) and *cytb* (70.2%), whereas *atp8* and *nad6* display the highest values (80.6% and 78.8%, respectively) (Supplementary Table S5; Supplementary Fig. S1B). The third codon of PCGs has a relatively higher A + T content, ranging from 63.1 % to 92.4 % with an average of 84.1 %, compared to the first and second codons (with a range of 59.9 % to 74.1 % and an average of 68.4 % and 67.7 %, respectively) (Supplementary Table S4; Supplementary Fig. S1B). Nucleotide-skew analyses showed that both AT- and GC-skews were negative in the family Curculionidae. The correlation between AT skew and A + T content of the mitochondrial genomes was significantly negative, indicating that a decrease in A + T contents would result in more significant AT skew. Similarly, a decrease in G + C contents would result in a more significant GC skew (Supplementary Table S6, Supplementary Fig. S1C and D).

There are 15 start codons for PCGs of mitogenomes in Curculionoidea, with ATT, ATA, ATG, and ATC being the most frequently used. The less common start codons include TTG, TTA and TCG, and the rest only appear once. Stop codons in these genes include TAG, TAA, and truncated T codons. ATG is commonly used as a start codon in *atp6*, *cox3*, *nad4*, *nad41*, and *cytb*. ATT always serves as the start codon in *nad2*, *cox*1, *atp8*, *nad5*, and *nad6*, whereas the remaining genes mostly start with either ATT or ATA. For all these genes except *cytb* and *nad1*, TAA is used as a stop codon slightly more frequently than TAG (Supplementary Table S7).

**Fig. 2.**
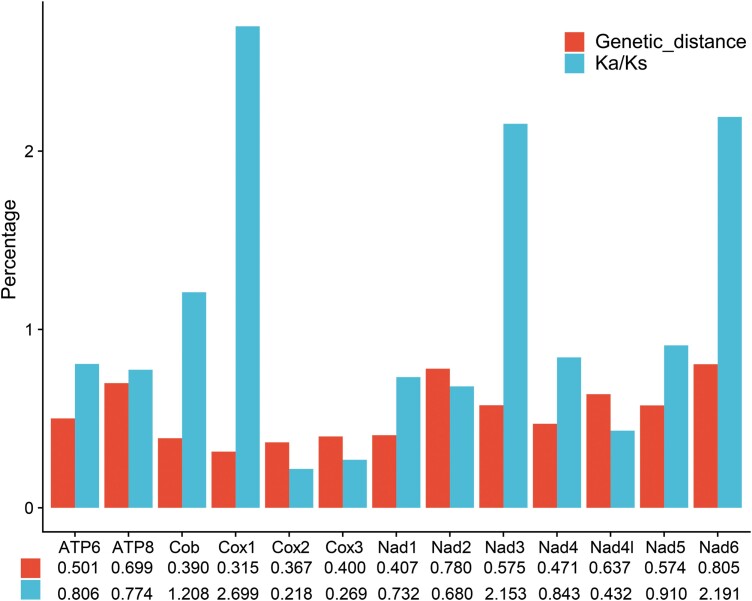
*K*_a_/*K*_s_ ratios and genetic distances for protein-coding genes. Leftside bars in each pair represent mean genetic distances; rightside bars in each pair represent mean *K*_*a*_/*K*_*s*_ (the ratio of the nonsynonymous replacement [*K*_*a*_] to the synonymous replacement [*K*_*s*_]) values; below each gene are the corresponding mean values.

**Fig. 3.**
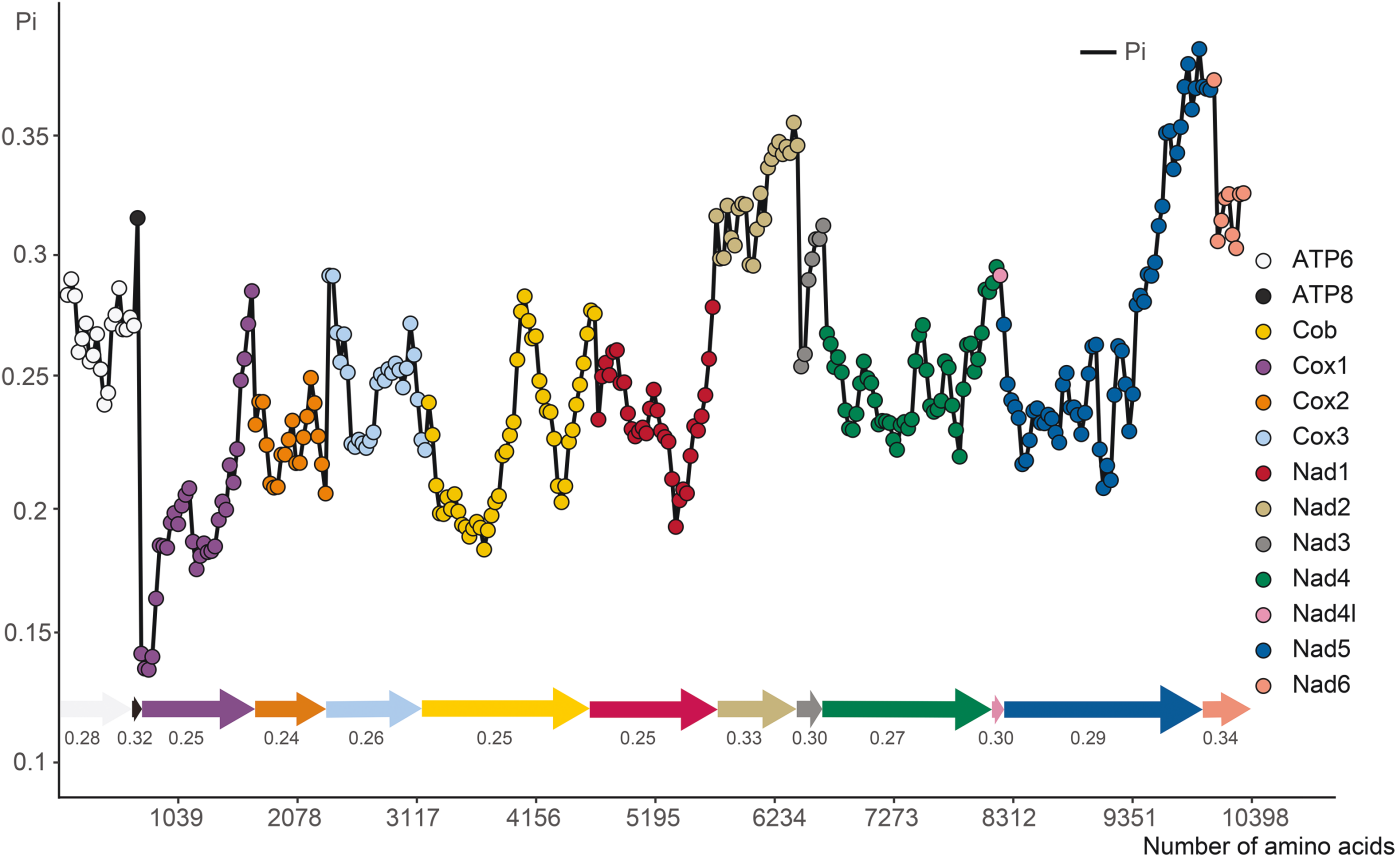
Nucleotide diversity (Pi) of PCGs of Curculionidae. Data represent the value of nucleotide diversity (a sliding window of 200 bp with a step size of 20 bp), and the mean value for each PCG is displayed below the corresponding points. The horizontal axis represents the number of amino acids for PCGs.

RSCU analysis revealed the preference of 22 synonymous codons in the mitogenomes of Curculionoidea. UUA-leu2 is the most frequently used codon, followed by UUU-Phe, GUU-Val, UCU-Ser, CCU-Pro, ACU-Thr, and UAU-Tyr (Supplementary Table S8). In addition, pairwise *K*_a_/*K*_s_ (*ω*) analyses showed that the *ω* values of *cob*, *cox1*, *nad3*, and *nad6* are > 1 (*ω* = 1.208, 2.699, 2.153, and 2.191, respectively), while the *ω* values of remaining 9 PCGs do not exceed 1 (Supplementary Table S9; Fig. 2). The mean pairwise-genetic-distance analyses revealed that *cytb* (0.390), *cox*1 (0.315), and *cox2* (0.367) have the lowest values, whereas *nad6* (0.805) and *atp8* (0.699) have the highest values (Supplementary Table S10; Fig. 2). The nucleotide diversity of 13 PCGs was analyzed with the sliding-window technique. The highest variability was found in *nad6* (Pi = 0.34), followed by *nad2* and *atp8* (Pi = 0.33 and 0.32). *Cox2* (Pi = 0.24) has the lowest variability (Supplementary Table S10; Fig. 3).

### Phylogenetic Relationships at Subfamily and Higher Levels

At the family level, all our phylogenetic analyses support 5 families (Cimberididae, Anthribidae, Attelabidae, Brentidae, and Curculionidae) of Curculionoidea as monophyletic groups and Cimberididae as the sister group of all other families, in agreement with the previous study of Shin et al. (2018). However, inconsistencies arose in the positions of Anthribidae and Attelabidae. When utilizing site-heterogeneity models [LG + PMSF (C60) and gamma] in conjunction with the PCGAA matrix, Attelabidae were inferred to be the sister group of the remaining 3 families (Anthribidae, Brentidae, and Curculionidae), whereas Anthribidae were supported by other models (i.e., partition, GHOST, and Bayes) as the second-splitting group. Despite the fact that the phylogenetic relationships among the subfamilies of Curculionidae remain unresolved under various models, the following conclusions can be drawn: (i) all analyses except for the ALL and PCGAA matrices supported the sister-group relationship of Dryophthorinae and Platypodinae, and the 2 as first splitting clade within the family Curculionidae (Supplementary Figs. S2–S11); (ii) the remaining subfamilies (except Bagoinae) were divided into 2 monophyletic groups (CEGH [Cyclominae, Entiminae, Gonipterini, Hyperinae] and CCCMS [Conoderinae, Cossoninae, Curculioninae, Molytinae, Scolytinae]); and (iii) in all datasets the sister-group relationship of Scolytinae and all remaining CCCMS clades was supported.

### Phylogenetic Inferences for Ceutorhynchini

**Fig. 4.**
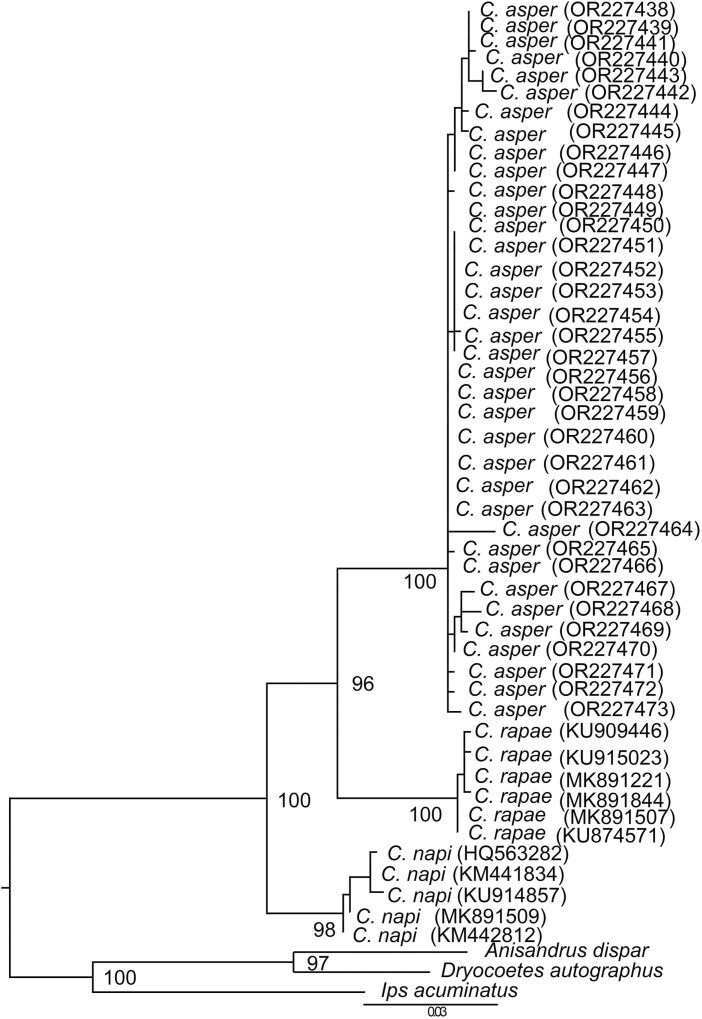
Phylogenetic tree constructed from the *cox1* (681 bp) dataset of *Ceutorhynchus napi* (5 samples), *Ceutorhynchus rapae* (6 samples), and *Ceutorhynchus asper* (25 samples). The numbers at the nodes represent bootstrap values. The numbers in parentheses represent the accession numbers.

**Fig. 5.**
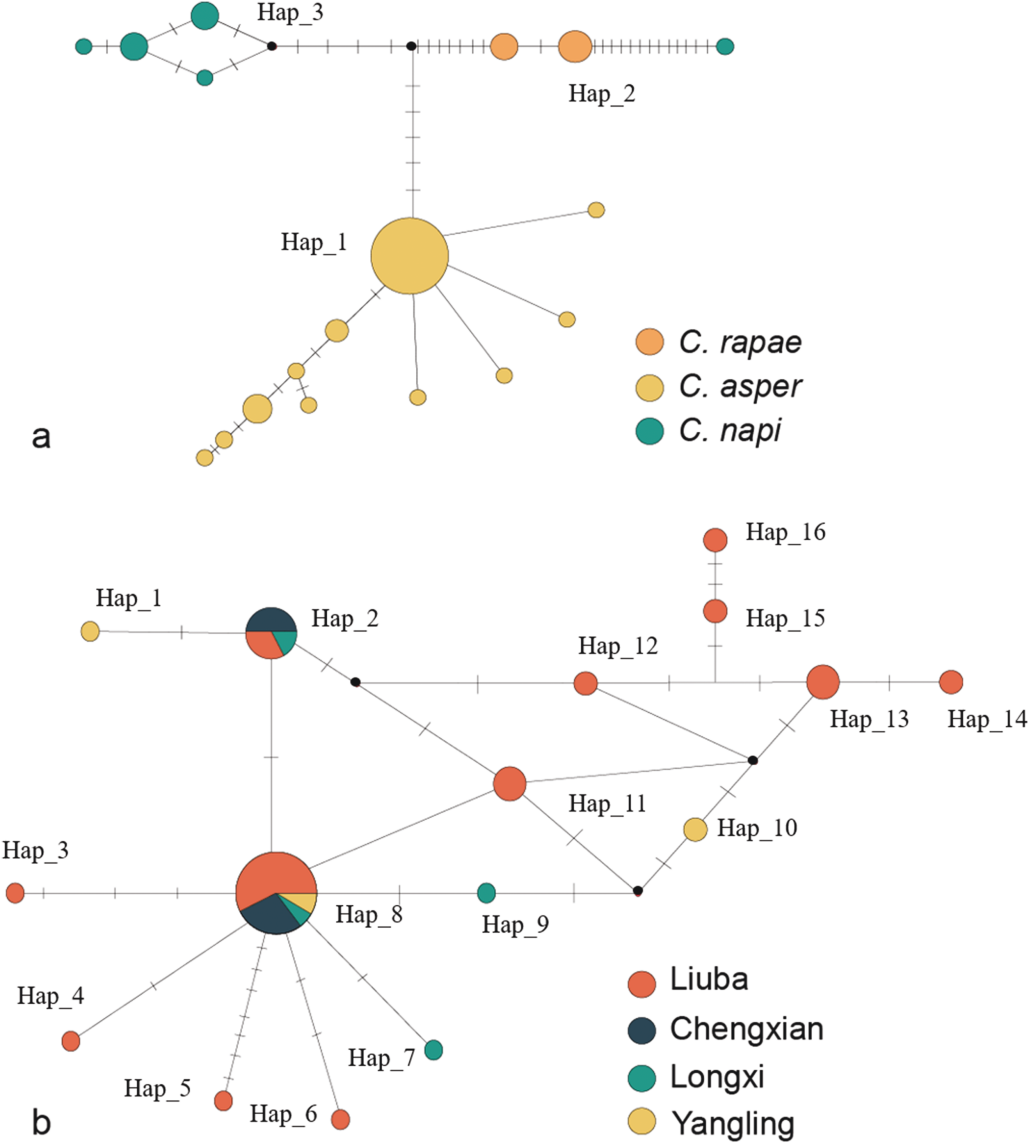
Median-joining network of cox1 haplotypes for 3 closely related *Ceutorhynchus* species. A) Cox1 haplotypes for *C. napi*, *C. rapae*, and *C. aspe*r. B) *Cox1* haplotypes for *C. asper* from different places (i.e., Chengxian, Liuba, Longxi, and Yangling). The size of each circle is proportional to the frequency of the haplotype. Black solid circles represent haplotypes without samples or being extinct, and dashes represent missing haplotypes.

Phylogenetic trees were also constructed using 48 trimmed *cox1* fragments from 3 species (*C. napi*, *C. rapae*, and *C. asper*). A total of 89 (1.3%) polymorphic nucleotide sites were identified in the *cox1* sequence of 681 bp, among which 65 were parsimony-informative. The trees recovered 3 separate clades for *C. napi*, *C. rapae*, and *C. asper*, with nodal support values of 98%, 99%, and 99%, respectively (Fig. 4). The mean intraspecific genetic distances between *cox*1 sequences of *C. napi*, *C. rapae*, and *C. asper* were 0.05%, 2.7%, and 0.05%, respectively, whereas all between-species genetic distances exceeded 6%, with an average of 6.4%. The pairwise F_ST_ values were 0.69 between *C. napi* and *C. rapae*, 0.91 between *C. napi* and *C. asper*, and 0.81 between *C. rapae* and *C. asper*, indicating high levels of genetic differentiation among the 3 species. Furthermore, the median-joining network analysis showed 18 haplotypes forming 3 distinct clusters (Fig. 5A). Among these clusters, *C. napi*, *C. rapae*, and *C. asper* contain 2, 5, and 11 haplotypes, respectively. These findings provide strong evidence that the 3 taxa represent distinct species entities.

In addition, haplotype analysis was separately conducted based on *cox1* of *C. asper* collected from different geographic locations in China (Supplementary Table S1). A total of 16 haplotypes were detected in the 4 populations (i.e., Chengxian, Liuba, Longxi, and Yangling). There were 14 unique haplotypes in the populations. The haplotype Hap_1 was shared by all 4 populations, and Hap_2 was found in 3 populations (all sampled populations except Yangling) (Fig. 5B). The nucleotide diversity (Pi) ranged from 0.0007 to 0.00403 for the 4 populations, and their haplotype diversities (Hd) were 0.500, 0.874, 0.786, and 0.833, respectively. The AMOVA showed that genetic variation mainly occurred within populations (76.7%), and there was no significant genetic differentiation among the 4 populations of different places.

**Fig. 6.**
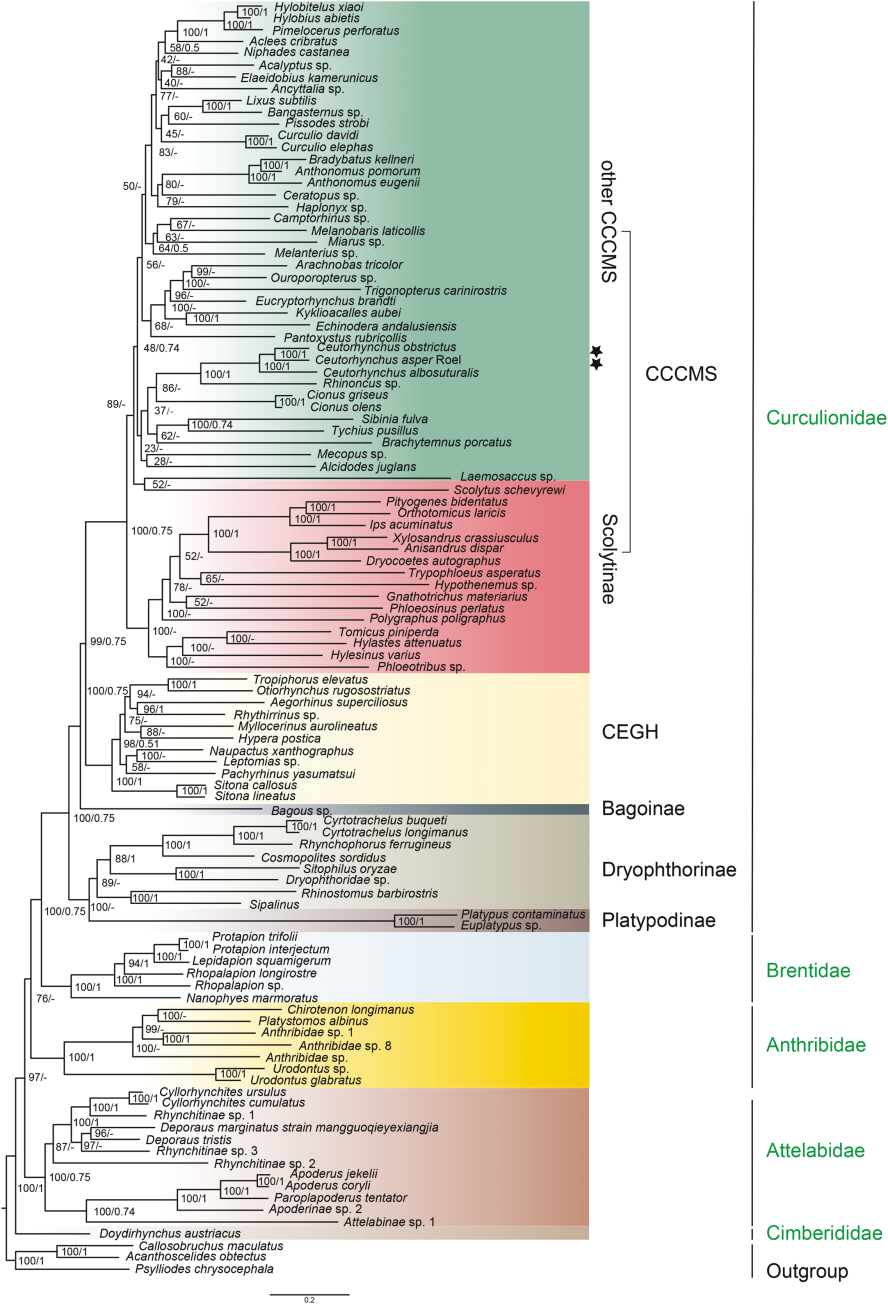
Phylogenetic tree inferred from the PCG matrix based on the site-heterogeneous model (LG + C60). The numbers at nodes show bootstrap values (BS)/posterior probabilities (PP); “‐” indicates the nodes unsupported by MrBayes analyses; black stars represent the 2 newly sequenced species of this study, showing their respective positions in the phylogenetic tree; the scale bar represents phylogenetic distance.

The phylogenetic position of *C. asper* and *C. albosuturalis* in Curculionoidea was examined by performing ML and Bayesian analyses of mitogenome sequences (Supplementary Tables S2 and S3). Analyses of all matrices (including PCG, PCG12, PCGAA, ALL, and SRH) with partitioning, GHOST and site-heterogeneous models [LG + PMSF (C60)] supported the position of *C. asper* and *C. albosuturalis* in the CCCMS clade of the family Curculionidae (Bayesian posterior probabilities [BPP] > 0.75 and bootstrap values (BS) > 95) (Fig. 6; Supplementary Tables S2–S11). *Homorosoma aspserum*, *C. albosuturalis*, *C. obstrictus*, and *Rhinoncus* sp. formed a monophyletic group with high node support values (BPP = 1 and BS = 100) (Fig. 6; Supplementary Tables S2–S11).

**Fig. 7.**
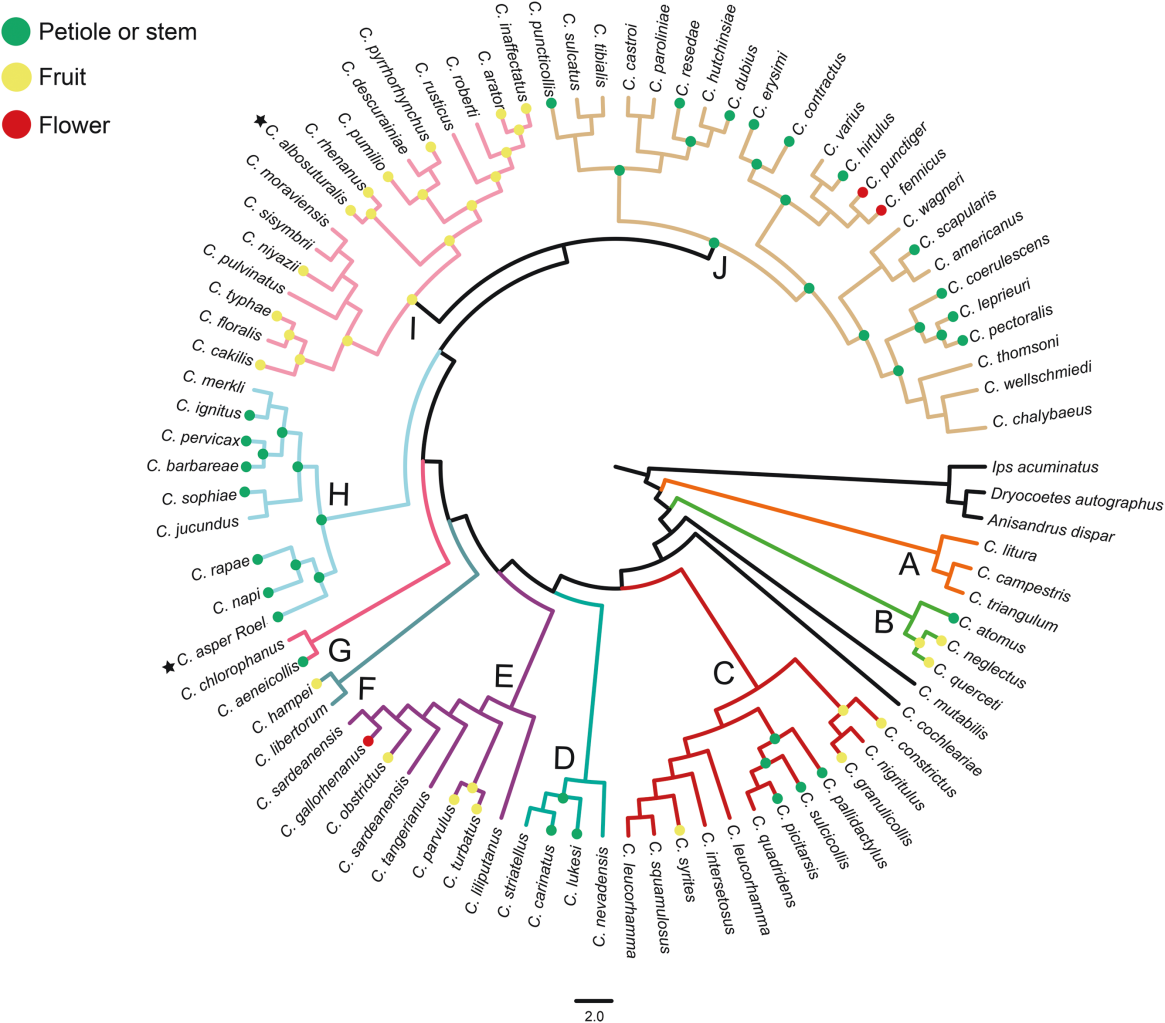
ML optimization of larval feeding niches conducted with Mesquite under the MK1 model of character evolution. Unmarked nodes represent unknown larval feeding niches or the equivocal ancestral status. Green, yellow, and red dots represent larval feeding niches of the stem (or petiole), fruit, and flower, respectively. Stars represent samples collected in this study.

To further determine the phylogenetic position of *H. aspserum* and *C. albosuturalis*, we conducted a phylogenetic analysis using 89 *cox1* sequences (Supplementary Table S2). The trimmed *cox*1 fragment had a length of 681 bp. The ML analysis generated 10 clades (A to J) for the genus *Ceutorhynchus* (with *C. asper* included), and strong statistical support for the major nodes suggests that these clades derived from ML analyses are reliable (Fig. 7). *Ceutorhynchus albosuturalis* falls in clade I, but *C. napi*, *C. rapae*, and *C. asper* all cluster in clade H. Ancestral state reconstruction suggested that the closest ancestors of clades H and I could occupy a niche of larvae feeding in host stems (or petioles) and fruits, respectively (Fig. 7). The niche of the closest ancestors of clade J was also reconstructed as larval feeding in stems (or petioles), while the niche of other clades of *Ceutorhynchus* was ambiguous.

## Discussion

### Mitogenome and Genetic Characterization of *C. asper* and *C. albosuturalis*

We assembled mitogenomes of the 2 Ceutorhynchine, *C. asper* and *C. albosuturalis*, and identified locations and orientations of PCGs, ribosomal RNA genes, and tRNA (except tRNA^Ile^) genes, consistent with previous studies (Cameron 2014). However, tRNA^Ile^ (also known as *trnI*) was found to be absent in both species. The frequent missing of *trnI* in published mitogenomes may be an artifact of mitogenome assembling since *trnI* is located in the control region, which is difficult to assemble (Song et al. 2010, Liu et al. 2016, Xu et al. 2017, Zhang et al. 2017, Yang et al. 2018). The 2 mitochondrial genomes analyzed in our study have relatively long nonencoding regions between tRNA^Ala^ and tRNA^Arg^ (*C. asper*: 501 bp, *C. albosuturalis*: 118 bp). This is consistent with previous studies of mitochondrial genomes in 5 subfamilies of Curculionidae, named Cryptorhynchinae, Curculioninae, Dryophthorinae, Molytinae, and Scolytinae (Song et al. 2020).

Weevils such as *C. asper*, *C. napi*, and *C. rapae* pose a serious threat to rapeseed crops in Europe and Asia (Li et al. 2009, Schaefer et al. 2017). They all primarily infest rapeseed plants and share similar life histories, although *C. napi* is geographically isolated from *C. rapae* and *C. asper* (Li et al. 2009, Schaefer et al. 2017). Furthermore, distinguishing the 3 species on morphological characteristics is challenging, and their taxonomic and phylogenetic relationships remain poorly understood. Prior to our study, no molecular data were available for *C. asper*, and *cox1* records are available only for *C. napi* and *C. rapae* on NCBI. We generated a sequence of mitochondrial genomes and the *cox1* gene of *C. asper* and *C. albosuturalis* for genetic identification and phylogenetic reconstruction. We found that the *cox1* sequences of *C. asper* are clearly different from those of *C. rapae* or *C. napi*, although the sequence similarity among the 3 was 94.94% (Supplementary Fig. S12). Both phylogenetic and haplotype analyses of the *cox1* region also supported the classification of 3 separate species (*C. napi*, *C. apae*, and *C. asper*). In the phylogenetic analyses, the 3 species groups have high nodal support values (>90%), with no support for cryptic entities in a particular group (Fig. 7). In addition, the phylogenetic analyses indicate that *C. rapae* and *C. asper* are the most closely related, and the results of haplotype analyses were consistent with those of the phylogenetic analysis (Fig. 5A). Current research is primarily focused on genetic comparisons of the 3 species, with limited attention given to descriptions and comparisons of their morphological characteristics. Future studies should further analyze and compare these aspects for verification purposes (Šedivý and Kocourek 1994, Milovac et al. 2017, Daum et al. 2023).

Both *C. napi* and *C. asper* (known as rape stem weevil in Europe and China, respectively) mainly damage oilseed rape, laying their eggs in the stems, while cabbage, broccoli, and wild cruciferous plants are the primary food sources of *C. rapae*, as reported in the literature (Büechi 1988, Li et al. 2009). In our ML analysis, *C. napi*, *C. rapae*, and *C. asper* cluster in the clade H, which may be attributed to convergent evolution due to their shared nutritional niche for cruciferous plants (Li et al. 2009). In our study, a closer relationship is found between *C. rapae* and *C. asper*, rather than between *C. asper* and *C. napi* (Fig. 4, 5A). *Ceutorhynchus napi* occurs mainly in Europe and North America and *C. rapae* across East Asia and Europe (Williams 2010, Vaitelyté and Brazauskiene 2012, Schaefer et al. 2017, Korotyaev 2021), whereas *C. asper* has only been reported to occur in China (Li et al. 2009). Interestingly, both *C. rapae* and *C. asper* occur in northwestern China, with similar geographical ranges (Li et al. 2009, Korotyaev 2021). Therefore, it is possible that the primary factor contributing to the differentiation between *C. napi* and the other 2 species is geographical isolation rather than nutritional niche.

### Phylogeny and Evolution of *Ceutorhynchus* Species and Related Weevils

The classification scheme of the family Curculionidae has been unstable, and no consistent results have been achieved by any scheme (Gunter et al. 2016, Song et al. 2020, Smit et al. 2022). However, the majority of our analyses with different data matrices and models support that Scolytinae is a monophyletic group and its sister-group relationships with the remaining CCCMS clades in Curculionidae. Our analyses also clearly show that Cyclominae, Entiminae, Gonipterini, and Hyperinae form a distinct clade and that Curculioninae, Conoderinae, Cossoninae, Molytinae, and Scolytinae coalesce into another clade, thus supporting 2 monophyletic groups (i.e., CEGH and CCCMS) as previously reported (Gillett et al. 2014, Gunter et al. 2016, Smit et al. 2022). *K*_a_/*K*_s_ ratios (*ω*) can serve as an indicator of purifying, neutral or positive selection in PCGs (McFerrin and Stone, 2011). In our analysis of PCGs, *cytb*, *cox*1, *nad3*, and *nad6* (*ω* > 1) show signs of positive selection with accelerated evolutionary rates (Fig. 2). As conserved loci are more suitable to be used as phylogenetic markers, it may be necessary to choose more slower evolving loci to address phylogenetic relationships in Curculionidae (McKenna et al. 2019).

In our phylogenetic analyses, we identify a relatively clear position of *C. asper* and *C. albosuturalis* in the family Curculionidae, based on mitochondrial genomes. To further determine the phylogenetic relationships of these 2 species with other species within the genus *Ceutorhynchus*, the available *cox1* was used as a phylogenetic marker to construct a tree of *Ceutorhynchus* species. Our results suggest that *C. asper* and *C. albosuturalis* fall in the clades H and I, respectively (Fig. 7). Interestingly, larvae of weevils in the clade H mainly feed and develop in the stem of cruciferous plants, whereas those in clade I mainly feed and develop in the fruits of the same host plants (Supplementary Table S11; Fig. 7). This observation makes sense, as different parts of the same host plant can provide different nutrients and microhabitats for herbivores, thus creating different ecological niches for herbivores and facilitating their versatile feeding and habitat colonization (Motta et al. 1995, Magalhães et al. 2017). The extraordinary diversity of weevils is usually attributed to their coevolution with angiosperms, and a significant factor driving this radiation is the specialization of weevil species onto specific plant tissues or specific plant species (Marvaldi et al. 2002, Oberprieler et al. 2007, Hernández-vera et al. 2010). Therefore, shifts in larval feeding behaviors and ecological niche specialization can play a critical role in the adaptive radiation of *Ceutorhynchus* species.

In summary, our study presents 2 new mitogenome sequences of *C. asper* and *C. albosuturalis*. Based on available mitogenome sequences of Curculionoidea and comprehensive phylogenetic analyses of 5 datasets, further evidence is provided to support monophyletic groups, including 5 families (i.e., Cimberididae, Anthribidae, Attelabidae, Brentidae, and Curculionidae), and 2 branches (i.e., CCCMS and CEGH) of Curculionidae. However, mitogenomes of more species in Curculionoidea are still needed in order to resolve subfamily and family-level relationships in this group. Both *C. asper* and *C. albosuturalis* belong to the CCCMS branch of Curculionidae, and they fall in clades H and I of *Ceutorhynchus*, respectively. Ancestral state reconstruction for *Ceutorhynchus* species shows that larvae in clades H and J mainly feed on stems (or petioles) of host plants, whereas those of clade I feed on fruits of the same host plants, suggesting that ecological niche specialization can play a critical role in the diversification of this group. In addition, based on the haplotype network analysis, *C. asper* showed little genetic differentiation across different altitudes and regions in the 2 provinces of Shaanxi and Gansu. There are few studies on the morphology, ecology, and physiology of *C. albosuturalis*, and further research is needed. Our data provide baseline molecular and genetic information for future research of *Ceutorhynchus* species and insights into the phylogeny and evolution of weevils in Curculionidae.

## Supplementary Material

ieae038_suppl_Supplementary_Tables_S1-S11_Figures_S1-S12

## Data Availability

DNA barcode sequences are available on GenBank (accession numbers: OR227438–OR227473), as well as the complete mitochondrial genomes (accession numbers: OR255927 and OR255928).
