# Supplementary material for: Genetic characterization of 2 Ceutorhynchus (Coleoptera: Curculionidae) weevils with mitogenomes and insights into the phylogeny and evolution of related weevils
Source: J Insect Sci. 2024 Mar 27;24(2):14. doi: 10.1093/jisesa/ieae038 (PMC10972553; doi:10.1093/jisesa/ieae038)
Supplement: ieae038_suppl_Supplementary_Tables_S1-S11_Figures_S1-S12 [file ieae038_suppl_supplementary_tables_s1-s11_figures_s1-s12.docx]

**Supporting Information**

**Genetic characterization of two *Ceutorhynchus* weevils with** **mitogenomes*,* and insights into the phylogeny and evolution of related weevils in** **Curculionidae**

**Table S1. Information for *Ceutorhynchus asper* specimens collected from Shaanxi and Gansu provinces in China**

| **Specimen** | **Collection Date** | **Region** | **Latitude** | **Longitude** | **Altitude** | **Host plant** |
| --- | --- | --- | --- | --- | --- | --- |
| YL1-1 | 2-Mar-22 | Yangling | 34.264528 | 108.061212 | 462.89 | Oilseed rape |
| YL1-2 | 2-Mar-22 | Yangling | 34.264528 | 108.061212 | 462.89 | Oilseed rape |
| YL1-3 | 2-Mar-22 | Yangling | 34.264528 | 108.061212 | 462.89 | Oilseed rape |
| YL1-4 | 2-Mar-22 | Yangling | 34.264528 | 108.061212 | 462.89 | Oilseed rape |
| LB5-1 | 5-Mar-23 | Nanzheng | 33.091493 | 106.845534 | 758.71 | Oilseed rape |
| LB5-2 | 5-Mar-23 | Nanzheng | 33.091493 | 106.845534 | 758.71 | Oilseed rape |
| LB5-3 | 5-Mar-23 | Nanzheng | 33.091493 | 106.845534 | 758.71 | Oilseed rape |
| LB5-4 | 5-Mar-23 | Nanzheng | 33.091493 | 106.845534 | 758.71 | Oilseed rape |
| LB2-1 | 6-Mar-22 | Liuba | 33.56316 | 106.974866 | 800.55 | cabbage |
| LB2-2 | 6-Mar-22 | Liuba | 33.56316 | 106.974866 | 800.55 | cabbage |
| LB2-3 | 6-Mar-22 | Liuba | 33.56316 | 106.974866 | 800.55 | cabbage |
| LB2-4 | 6-Mar-22 | Liuba | 33.56316 | 106.974866 | 800.55 | cabbage |
| LB1-1 | 6-Mar-22 | Liuba | 33.722845 | 107.062671 | 869.84 | Oilseed rape |
| LB1-2 | 6-Mar-22 | Liuba | 33.722845 | 107.062671 | 869.84 | Oilseed rape |
| LB1-3 | 6-Mar-22 | Liuba | 33.722845 | 107.062671 | 869.84 | Oilseed rape |
| LB1-4 | 6-Mar-22 | Liuba | 33.722845 | 107.062671 | 869.84 | Oilseed rape |
| LB3-1 | 6-Mar-22 | Liuba | 33.62018 | 106.910668 | 983.59 | Shepherd's purse |
| LB3-2 | 6-Mar-22 | Liuba | 33.62018 | 106.910668 | 983.59 | Shepherd's purse |
| LB3-3 | 6-Mar-22 | Liuba | 33.62018 | 106.910668 | 983.59 | Shepherd's purse |
| LB3-4 | 6-Mar-22 | Liuba | 33.62018 | 106.910668 | 983.59 | Shepherd's purse |
| LB4-1 | 6-Mar-22 | Liuba | 33.62018 | 106.910668 | 983.59 | Oilseed rape |
| LB4-2 | 6-Mar-22 | Liuba | 33.62018 | 106.910668 | 983.59 | Oilseed rape |
| LB4-3 | 6-Mar-22 | Liuba | 33.62018 | 106.910668 | 983.59 | Oilseed rape |
| LB4-4 | 6-Mar-22 | Liuba | 33.62018 | 106.910668 | 983.59 | Oilseed rape |
| CX1-1 | 21-Mar-23 | Chengxian | 33.772619 | 105.846772 | 1066.3 | Oilseed rape |
| CX1-2 | 21-Mar-23 | Chengxian | 33.772619 | 105.846772 | 1066.3 | Oilseed rape |
| CX1-3 | 21-Mar-23 | Chengxian | 33.772619 | 105.846772 | 1066.3 | Oilseed rape |
| CX1-4 | 21-Mar-23 | Chengxian | 33.772619 | 105.846772 | 1066.3 | Oilseed rape |
| LX2-1 | 15-Apr-23 | Longxi | 34.89206 | 104.861267 | 1989.64 | Oilseed rape |
| LX2-2 | 15-Apr-23 | Longxi | 34.89206 | 104.861267 | 1989.64 | Oilseed rape |
| LX2-3 | 15-Apr-23 | Longxi | 34.89206 | 104.861267 | 1989.64 | Oilseed rape |
| LX2-4 | 15-Apr-23 | Longxi | 34.89206 | 104.861267 | 1989.64 | Oilseed rape |
| LX1-1 | 15-Apr-23 | Longxi | 34.90316 | 104.519541 | 2114.76 | Oilseed rape |
| LX1-2 | 15-Apr-23 | Longxi | 34.90316 | 104.519541 | 2114.76 | Oilseed rape |
| LX1-3 | 15-Apr-23 | Longxi | 34.90316 | 104.519541 | 2114.76 | Oilseed rape |
| LX1-4 | 15-Apr-23 | Longxi | 34.90316 | 104.519541 | 2114.76 | Oilseed rape |

**Table S2. DNA barcoding (*cox1*) sequences downloaded from GenBank of NCBI**

| **Species** | **GenBank accession** | **Species** | **GenBank**  **accession** | **Species** | **GenBank**  **accession** |
| --- | --- | --- | --- | --- | --- |
| *Anisandrus dispar* | ON164665 | *Ceutorhynchus nigritulus* | MK891118 | *Ceutorhynchus napi* | HQ563282 |
| *Dryocoetes autographus* | NC_036287 | *Ceutorhynchus niyazii* | MH218861 | *Ceutorhynchus napi* | KM441834 |
| *Ips acuminatus* | OL546657 | *Ceutorhynchus obstrictus* | KU912389 | *Ceutorhynchus napi* | MK891509 |
| *Ceutorhynchus aeneicollis* | EU110945 | *Ceutorhynchus pallidactylus* | KU366275 | *Ceutorhynchus napi* | KU914857 |
| *Ceutorhynchus americanus* | KC783939 | *Ceutorhynchus paroliniae* | KM446446 | *Ceutorhynchus napi* | KM442812 |
| *Ceutorhynchus arator* | EU110947 | *Ceutorhynchus parvulus* | MK891559 | *Ceutorhynchus merkli* | MK891375 |
| *Ceutorhynchus atomus* | KM450470 | *Ceutorhynchus pectoralis* | MK890987 | *Ceutorhynchus moraviensis* | MN249774 |
| *Ceutorhynchus barbareae* | KC783969 | *Ceutorhynchus pervicax* | EU110992 | *Ceutorhynchus mutabilis* | KM441834 |
| *Ceutorhynchus cakilis* | MZ633598 | *Ceutorhynchus picitarsis* | KC783950 | *Ceutorhynchus napi* | DQ058688 |
| *Ceutorhynchus campestris* | KC783937 | *Ceutorhynchus pulvinatus* | HQ954146 | *Ceutorhynchus neglectus* | MK891249 |
| *Ceutorhynchus carinatus* | MN729499 | *Ceutorhynchus pumilio* | MK891116 | *Ceutorhynchus nevadensis* | MK891883 |
| *Ceutorhynchus castroi* | MK891181 | *Ceutorhynchus puncticollis* | HQ559232 | *Ceutorhynchus rapae* | KU909446 |
| *Ceutorhynchus cf._chalybaeus* | MK891496 | *Ceutorhynchus punctiger* | KU916783 | *Ceutorhynchus rapae* | KU915023 |
| *Ceutorhynchus cf._erysimi* | MK891173 | *Ceutorhynchus pyrrhorhynchus* | HQ954276 | *Ceutorhynchus rapae* | MK891221 |
| *Ceutorhynchus cf._leucorhamma* | KC783872 | *Ceutorhynchus quadridens* | KJ963926 | *Ceutorhynchus rapae* | MK891844 |
| *Ceutorhynchus cf._sardeanensis* | EU110948 | *Ceutorhynchus querceti* | KU915023 | *Ceutorhynchus rapae* | MK891507 |
| *Ceutorhynchus chalybaeus* | KC783929 | *Ceutorhynchus resedae* | HQ953920 | *Ceutorhynchus rapae* | KU874571 |
| *Ceutorhynchus chlorophanus* | EU110952 | *Ceutorhynchus rhenanus* | EU110997 | *Ceutorhynchus libertorum* | MK891236 |
| *Ceutorhynchus cochleariae* | MK891548 | *Ceutorhynchus roberti* | HQ953854 | *Ceutorhynchus liliputanus* | KJ966320 |
| *Ceutorhynchus coerulescens* | KM447569 | *Ceutorhynchus rusticus* | MK891177 | *Ceutorhynchus litura* | MK891187 |
| *Ceutorhynchus constrictus* | KC784315 | *Ceutorhynchus sardeanensis* | JF889905 | *Ceutorhynchus lukesi* | MK891205 |
| *Ceutorhynchus contractus* | MK890854 | *Ceutorhynchus scapularis* | EU551169 | *Ceutorhynchus rapae* | KM446998 |
| *Ceutorhynchus descurainiae* | KM445661 | *Ceutorhynchus sisymbrii* | MK891549 | *Ceutorhynchus rapae* | EU110995 |
| *Ceutorhynchus dubius* | KJ963154 | *Ceutorhynchus sophiae* | MK891843 | *Ceutorhynchus rapae* | EU110996 |
| *Ceutorhynchus erysimi* | KM450774 | *Ceutorhynchus squamulosus* | EU110998 | *Ceutorhynchus rapae* | KJ963893 |
| *Ceutorhynchus fennicus* | AY837617 | *Ceutorhynchus striatellus* | AY692339 | *Ceutorhynchus jucundus* | KC784260 |
| *Ceutorhynchus floralis* | KJ963770 | *Ceutorhynchus sulcatus* | EU110999 | *Ceutorhynchus leprieuri* | MK891175 |
| *Ceutorhynchus gallorhenanus* | MK891233 | *Ceutorhynchus sulcicollis* | MK891508 | *Ceutorhynchus leucorhamma* | MK890848 |
| *Ceutorhynchus granulicollis* | KC783926 | *Ceutorhynchus syrites* | MK891170 | *Ceutorhynchus varius* | KC783930 |
| *Ceutorhynchus hampei* | KM440763 | *Ceutorhynchus tangerianus* | KJ963985 | *Ceutorhynchus wagneri* | MK891521 |
| *Ceutorhynchus hirtulus* | KC783914 | *Ceutorhynchus thomsoni* | MK891172 | *Ceutorhynchus wellschmiedi* | KJ846078 |
| *Ceutorhynchus hutchinsiae* | KC783948 | *Ceutorhynchus tibialis* | KJ962289 | *Ceutorhynchus inaffectatus* | EU110966 |
| *Ceutorhynchus ignitus* | HQ953999 | *Ceutorhynchus triangulum* | KM448854 | *Ceutorhynchus intersetosus* | KC783783 |
| *Ceutorhynchus typhae* | KC783932 | *Ceutorhynchus turbatus* | MK891150 |  |  |

**Table S3. Information for taxa and the *cox1* accession numbers used in this study**

| **Family** | **Subfamily** | **Tribe** | **Species** | **GenBank accession** |
| --- | --- | --- | --- | --- |
| Curculionidae | Bagoinae | Bagoini | *Bagous* sp. | MH404106.1 |
| Curculionidae | Conoderinae | Mecopini | *Mecopus* sp. | MH431911.1 |
| Curculionidae | Conoderinae | Baridini | *Melanobaris laticollis* | JN163955.1 |
| Curculionidae | Cossoninae | Onycholipini | *Brachytemnus porcatus* | JN163960.1 |
| Curculionidae | Curculioninae | Acalyptini | *Acalyptus* sp. | MH404107.1 |
| Curculionidae | Curculioninae | Anthonomini | *Bradybatus kellneri* | KX087247.1 |
| Curculionidae | Curculioninae | Anthonomini | *Anthonomus eugenii* | NC_044711.1 |
| Curculionidae | Curculioninae | Anthonomini | *Anthonomus pomorum* | NC_044712.1 |
| Curculionidae | Curculioninae | Ceratopodini | *Ceratopus* sp. | MH473535.1 |
| Curculionidae | Curculioninae | Ceutorhynchini | *Rhinoncus* sp. | MH404119.1 |
| Curculionidae | Curculioninae | Ceutorhynchini | *Ceutorhynchus asper* | OR255927 |
| Curculionidae | Curculioninae | Ceutorhynchini | *Ceutorhynchus albosuturalis* | OR255928 |
| Curculionidae | Curculioninae | Ceutorhynchini | *Ceutorhynchus obstrictus* | JN163956.1 |
| Curculionidae | Curculioninae | Cionini | *Cionus griseus* | MH404125.1 |
| Curculionidae | Curculioninae | Cionini | *Cionus olens* | JN163958.1 |
| Curculionidae | Curculioninae | Cleogonini | *Pantoxystus rubricollis* | KX087330.1 |
| Curculionidae | Curculioninae | Cleogonini | *Melanterius* sp. | MH404113.1 |
| Curculionidae | Curculioninae | Cryptoplini | *Haplonyx* sp. | MH431909.1 |
| Curculionidae | Curculioninae | Curculionini | *Curculio elephas* | KX087269.1 |
| Curculionidae | Curculioninae | Curculionini | *Curculio davidi* | NC_034293.1 |
| Curculionidae | Curculioninae | Derelomini | *Elaeidobius kamerunicus* | NC_049880.1 |
| Curculionidae | Curculioninae | Eugnomini | *Ancyttalia* sp. | MH404121.1 |
| Curculionidae | Curculioninae | Mecinini | *Miarus* sp. | MH404105.1 |
| Curculionidae | Curculioninae | Tychiini | *Sibinia fulva* | MH404129.1 |
| Curculionidae | Curculioninae | Tychiini | *Tychius pusillus* | MK692568.1 |
| Curculionidae | Cyclominae | Aterpini | *Aegorhinus superciliosus* | NC_027577.1 |
| Curculionidae | Cyclominae | Rhythirrinini | *Rhythirrinus* sp. | MH404126.1 |
| Curculionidae | Dryophthorinae | - | *Cyrtotrachelus buqueti* | MK682381.1 |
| Curculionidae | Dryophthorinae | - | *Cyrtotrachelus longimanus* | MK682380.1 |
| Curculionidae | Dryophthorinae | - | *Dryophthoridae* sp. | MH404101.1 |
| Curculionidae | Dryophthorinae | - | *Cosmopolites sordidus* | MH281567.1 |
| Curculionidae | Dryophthorinae | - | *Rhinostomus barbirostris* | MG193443.1 |
| Curculionidae | Dryophthorinae | Rhynchophorini | *Rhynchophorus ferrugineus* | CM025099.1 |
| Curculionidae | Dryophthorinae | Rhynchophorini | *Sitophilus oryzae* | NC_030765.1 |
| Curculionidae | Dryophthorinae | Orthognathini | *Sipalinus gigas* | NC_053351.1 |
| Curculionidae | Entiminae | Byrsopagini | *Tropiphorus elevatus* | KX087368.1 |
| Curculionidae | Entiminae | Cyphicerini | *Myllocerinus aurolineatus* | NC_040931.1 |
| Curculionidae | Entiminae | Naupactini | *Naupactus xanthographus* | NC_018354.1 |
| Curculionidae | Entiminae | Otiorhynchini | *Otiorhynchus rugosostriatus* | JN163969.1 |
| Curculionidae | Entiminae | Polydrusini | *Pachyrhinus yasumatsui* | MF807224.1 |
| Curculionidae | Entiminae | Sitonini | *Sitona lineatus* | JN163948.1 |
| Curculionidae | Entiminae | Sitonini | *Sitona callosus* | MF594624.1 |
| Curculionidae | Entiminae | Tanymecini | *Leptomias* sp. | MT536938.1 |
| Curculionidae | Hyperinae | Hyperini | *Hypera postica* | MK692605.1 |
| Curculionidae | Molytinae | Aminyopini | *Niphades castanea* | MT232762.1 |
| Curculionidae | Molytinae | Cryptorhynchini | *Arachnobas tricolor* | KX087241.1 |
| Curculionidae | Molytinae | Cryptorhynchini | *Ouroporopterus* sp. | MH404099.1 |
| Curculionidae | Molytinae | Cryptorhynchini | *Kyklioacalles aubei* | JN163957.1 |
| Curculionidae | Molytinae | Cryptorhynchini | *Trigonopterus carinirostris* | NC_050891.1 |
| Curculionidae | Molytinae | Cryptorhynchini | *Echinodera andalusiensis* | MK692645.1 |
| Curculionidae | Molytinae | Cryptorhynchini | *Eucryptorhynchus brandti* | NC_025945.1 |
| Curculionidae | Molytinae | Hylobiini | *Pimelocerus perforatus* | NC_053826.1 |
| Curculionidae | Molytinae | Hylobiini | *Hylobius abietis* | JN163954.1 |
| Curculionidae | Molytinae | Hylobiini | *Hylobitelus xiaoi* | NC_022680.1 |
| Curculionidae | Molytinae | Hylobiini | *Aclees cribratus* | NC_051548.1 |
| Curculionidae | Molytinae | Ithyporini | *Camptorhinus* sp. | MH404122.1 |
| Curculionidae | Molytinae | Lixini | *Lixus subtilis* | MW413392.1 |
| Curculionidae | Molytinae | Lixini | *Bangasternus* sp. | MH404135.1 |
| Curculionidae | Molytinae | Mecysolobini | *Alcidodes juglans* | NC_041669.1 |
| Curculionidae | Molytinae | Mesoptiliini | *Laemosaccus* sp. | MH404116.1 |
| Curculionidae | Molytinae | Pissodini | *Pissodes strobi* | MW452482.1 |
| Curculionidae | Platypodinae | Platypodini | *Euplatypus* sp. | KX035180.1 |
| Curculionidae | Platypodinae | Platypodini | *Platypus contaminatus* | NC_045889.1 |
| Curculionidae | Scolytinae | Corthylini | *Gnathotrichus materiarius* | NC_036294.1 |
| Curculionidae | Scolytinae | Cryphalini | *Hypothenemus* sp. | KX035163.1 |
| Curculionidae | Scolytinae | Cryphalini | *Trypophloeus asperatus* | NC_036285.1 |
| Curculionidae | Scolytinae | Dryocoetini | *Dryocoetes autographus* | NC_036287.1 |
| Curculionidae | Scolytinae | Hylastini | *Hylastes attenuatus* | NC_036290.1 |
| Curculionidae | Scolytinae | Hylesinini | *Hylesinus varius* | MH281571.1 |
| Curculionidae | Scolytinae | Hylurgini | *Tomicus piniperda* | MH281570.1 |
| Curculionidae | Scolytinae | Ipini | *Ips acuminatus* | MK988441.1 |
| Curculionidae | Scolytinae | Ipini | *Orthotomicus laricis* | NC_036291.1 |
| Curculionidae | Scolytinae | Ipini | *Pityogenes bidentatus* | NC_036289.1 |
| Curculionidae | Scolytinae | Phloeosinini | *Phloeosinus perlatus* | KX035210.1 |
| Curculionidae | Scolytinae | Phloeotribini | *Phloeophthorus* sp. | NC_057470.1 |
| Curculionidae | Scolytinae | Polygraphini | *Polygraphus poligraphus* | MN528600.1 |
| Curculionidae | Scolytinae | Scolytini | *Scolytus schevyrewi* | NC_046589.1 |
| Curculionidae | Scolytinae | Xyleborini | *Anisandrus dispar* | NC_036293.1 |
| Curculionidae | Scolytinae | Xyleborini | *Xylosandrus crassiusculus* | NC_036284.1 |
| Anthribidae | Anthribinae | Platystomini | *Platystomos albinus* | JN163968.1 |
| Anthribidae | Urodontinae | - | *Urodontus* sp. | JX412764.1 |
| Anthribidae | Anthribinae | Ecelonerini | *Chirotenon longimanus* | JX412830.1 |
| Anthribidae | Urodontinae | - | *Urodontus glabratus* | JX412859.1 |
| Anthribidae | - | - | *Anthribidae* sp. 1 | MH404103.1 |
| Anthribidae | - | - | *Anthribidae* sp. 8 | MH751301.1 |
| Anthribidae | - | - | *Anthribidae* sp. 6 | MH751306.1 |
| Brentidae | - | - | *Lepidapion squamigerum* | MN459662.1 |
| Brentidae | - | - | *Protapion interjectum* | MK692665.1 |
| Brentidae | - | - | *Protapion trifolii* | MK692609.1 |
| Brentidae | - | - | *Rhopalapion longirostre* | JN163967.1 |
| Brentidae | Nanophyinae | - | *Nanophyes marmoratus* | JN163946.1 |
| Attelabidae | Apoderinae | - | *Apoderinae* sp. | MH473531.1 |
| Attelabidae | Apoderinae | - | *Apoderus coryli* | OU452218.2 |
| Attelabidae | Apoderinae | - | *Apoderus jekelii* | MK292540.1 |
| Attelabidae | - | - | *Attelabinae* sp. 1 | MH404127.1 |
| Attelabidae | Rhynchitinae | - | *Cyllorhynchites cumulatus* | MZ027640.1 |
| Attelabidae | Rhynchitinae | - | *Cyllorhynchites ursulus* | MH156809.1 |
| Attelabidae | Rhynchitinae | - | *Deporaus marginatus strain mangguoqieyexiangjia* | NC 051943.1 |
| Attelabidae | Rhynchitinae | - | *Deporaus tristis* | KX087280.1 |
| Attelabidae | Apoderinae | - | *Paroplapoderus tentator* | MT113120.1 |
| Attelabidae | - | - | *Rhynchitinae* sp. 1 | MH751312.1 |
| Attelabidae | - | - | *Rhynchitinae* sp. 2 | MH751315.1 |
| Attelabidae | - | - | *Rhynchitinae* sp. 3 | MH751299.1 |
| Nemonychidae | - | - | *Doydirhynchus austriacus* | JN163964.1 |
| Chrysomelidae | Bruchinae | Bruchini | *Acanthoscelides_obtectus* | MF925724.1 |
| Chrysomelidae | Bruchinae | Bruchini | *Callosobruchus_maculatus* | KY942062.1 |
| Chrysomelidae | Galerucinae | Alticini | *Psylliodes_chrysocephala* | OU815750.1 |

**Table S4. AT contents of mitochondrial genes**

| **Species** | **rRNA** | **tRNA** | **PCG** | **Codon position of PCG** | | | **Full mitogenome** |
| --- | --- | --- | --- | --- | --- | --- | --- |
|  | AT% | AT% | AT% | 1 | 2 | 3 | AT% |
| *Acalyptus* sp. | 79.8 | 76.7 | 74.9 | 70.0 | 68.1 | 86.5 | 74.7 |
| *Acanthoscelides obtectus* | 81.2 | 77.9 | 74.2 | 69.9 | 68.5 | 84.3 | 74.5 |
| *Aclees cribratus* | 79.6 | 78.1 | 74.6 | 69.0 | 68.2 | 86.7 | 74.8 |
| *Aegorhinus superciliosus* | 78.1 | 78.0 | 74.6 | 69.0 | 67.6 | 87.2 | 74.6 |
| *Alcidodes juglans* | 75.8 | 76.0 | 74.0 | 68.2 | 68.0 | 85.9 | 73.8 |
| *Ancyttalia* sp. | 80.9 | 79.4 | 75.8 | 70.9 | 68.5 | 88.0 | 76.0 |
| *Anisandrus dispar* | 71.3 | 75.3 | 71.1 | 66.6 | 66.3 | 80.4 | 71.2 |
| *Anthonomus eugenii* | 76.2 | 75.7 | 71.0 | 67.4 | 67.2 | 78.3 | 71.2 |
| *Anthonomus pomorum* | 76.8 | 75.2 | 73.0 | 67.6 | 67.5 | 83.8 | 73.0 |
| *Anthribidae* sp. | 78.6 | 77.3 | 73.0 | 68.3 | 68.4 | 82.4 | 73.4 |
| *Anthribidae* sp. 1 | 83.5 | 80.5 | 78.4 | 72.7 | 69.8 | 92.8 | 78.6 |
| *Anthribidae* sp. 8 | 80.0 | 79.3 | 73.7 | 69.8 | 68.5 | 82.8 | 74.3 |
| *Apoderinae* sp. 2 | 76.1 | 75.4 | 66.9 | 62.9 | 65.5 | 72.5 | 67.8 |
| *Apoderus coryli* | 75.6 | 72.5 | 69.1 | 65.6 | 65.4 | 76.3 | 69.5 |
| *Apoderus jekelii* | 75.5 | 73.4 | 70.2 | 66.5 | 65.6 | 78.4 | 70.4 |
| *Arachnobas tricolor* | 76.4 | 79.6 | 71.9 | 68.7 | 68.3 | 78.7 | 72.5 |
| *Attelabinae* sp. 1 | 74.9 | 74.6 | 67.0 | 64.5 | 65.5 | 71.0 | 67.8 |
| *Bagous* sp. | 75.2 | 74.3 | 68.8 | 63.8 | 66.1 | 76.6 | 69.4 |
| *Bangasternus* sp. | 79.2 | 77.9 | 76.2 | 71.0 | 68.7 | 88.8 | 76.0 |
| *Brachytemnus porcatus* | 69.4 | 77.3 | 72.8 | 66.5 | 66.9 | 84.9 | 73.0 |
| *Bradybatus kellneri* | 76.6 | 77.5 | 72.9 | 68.4 | 68.0 | 82.3 | 73.1 |
| *Callosobruchus maculatus* | 80.0 | 77.8 | 73.3 | 68.9 | 68.3 | 82.7 | 73.7 |
| *Camptorhinus* sp. | 79.6 | 81.1 | 76.8 | 71.2 | 69.1 | 90.1 | 76.9 |
| *Ceratopus* sp. | 78.8 | 76.8 | 74.7 | 68.3 | 68.1 | 87.8 | 74.6 |
| *Ceutorhynchus obstrictus* | 72.9 | 77.7 | 76.0 | 70.3 | 69.0 | 88.8 | 75.9 |
| *Chirotenon longimanus* | 78.0 | 78.8 | 74.1 | 67.9 | 69.0 | 85.4 | 74.3 |
| *Cionus griseus* | 80.5 | 78.4 | 75.9 | 71.2 | 68.9 | 87.6 | 75.7 |
| *Cionus olens* | 76.2 | 78.0 | 75.9 | 70.6 | 69.3 | 87.7 | 75.8 |
| *Cosmopolites sordidus* | 75.3 | 79.1 | 73.0 | 67.5 | 66.8 | 84.7 | 73.3 |
| *Cryphalus abietis* | 73.5 | 74.4 | 71.6 | 66.2 | 66.7 | 81.9 | 75.5 |
| *Curculio davidi* | 76.7 | 76.7 | 75.9 | 70.7 | 68.4 | 88.7 | 76.0 |
| *Curculio elephas* | 78.0 | 78.9 | 76.1 | 70.7 | 68.4 | 89.2 | 71.4 |
| *Cyllorhynchites cumulatus* | 78.3 | 75.2 | 71.0 | 67.0 | 66.8 | 79.3 | 70.1 |
| *Cyllorhynchites ursulus* | 77.4 | 75.6 | 69.3 | 66.2 | 66.3 | 75.5 | 72.4 |
| *Cyrtotrachelus buqueti* | 76.1 | 76.3 | 72.2 | 66.9 | 66.5 | 83.3 | 72.7 |
| *Cyrtotrachelus longimanus* | 77.0 | 76.0 | 72.5 | 67.6 | 66.4 | 83.5 | 74.8 |
| *Deporaus marginatus strain mangguoqieyexiangjia* | 79.5 | 77.4 | 74.6 | 68.7 | 67.1 | 88.0 | 73.5 |
| *Deporaus tristis* | *-* | 76.6 | 73.4 | 67.8 | 67.3 | 85.1 | 77.3 |
| *Doydirhynchus austriacus* | 71.5 | 80.7 | 77.3 | 71.8 | 69.0 | 91.1 | 73.0 |
| *Dryocoetes autographus* | 74.5 | 76.9 | 72.6 | 67.4 | 67.0 | 83.5 | 73.1 |
| *Dryophthoridae* sp. | 75.8 | 76.9 | 73.2 | 68.8 | 67.7 | 83.1 | 71.3 |
| *Echinodera andalusiensis* | 77.5 | 77.3 | 70.8 | 67.2 | 68.2 | 77.0 | 72.4 |
| *Elaeidobius kamerunicus* | 77.7 | 77.1 | 72.0 | 67.5 | 67.7 | 80.9 | 76.0 |
| *Eucryptorhynchus brandti* | 78.4 | 78.1 | 76.2 | 69.4 | 68.5 | 90.6 | 73.6 |
| *Euplatypus* sp. | 79.6 | 77.5 | 73.3 | 68.3 | 67.5 | 84.2 | 64.0 |
| *Gnathotrichus materiarius* | 72.6 | 68.6 | 63.3 | 59.9 | 63.7 | 66.4 | 72.2 |
| *Haplonyx* sp. | 77.6 | 78.0 | 71.7 | 67.0 | 68.2 | 79.9 | 72.2 |
| *Hylastes attenuatus* | 78.4 | 76.6 | 71.8 | 68.1 | 67.2 | 80.2 | 78.3 |
| *Hylesinus varius* | 80.5 | 81.2 | 78.4 | 73.0 | 69.3 | 92.9 | 74.1 |
| *Hylobitelus xiaoi* | 79.2 | 77.0 | 74.0 | 68.8 | 67.7 | 85.5 | 73.9 |
| *Hylobius abietis* | 72.2 | 76.7 | 73.9 | 68.0 | 68.5 | 85.2 | 75.6 |
| *Hypera postica* | 77.9 | 78.3 | 75.8 | 69.4 | 67.6 | 90.2 | 69.0 |
| *Hypothenemus* sp. | 75.0 | 74.9 | 68.3 | 63.8 | 66.1 | 75.2 | 75.2 |
| *Ips acuminatus* | 80.1 | 78.4 | 74.9 | 69.1 | 67.4 | 88.3 | 76.7 |
| *Kyklioacalles aubei* | 70.0 | 79.0 | 76.9 | 71.7 | 70.2 | 88.9 | 76.6 |
| *Laemosaccus* sp. | 81.6 | 80.8 | 76.4 | 71.4 | 69.7 | 88.0 | 75.7 |
| *Lepidapion squamigerum* | 80.4 | 77.9 | 75.8 | 70.8 | 68.7 | 88.0 | 72.1 |
| *Leptomias* sp. | 75.5 | 76.4 | 71.9 | 66.2 | 67.4 | 82.1 | 74.9 |
| *Lixus subtilis* | 78.9 | 77.0 | 75.0 | 69.4 | 68.5 | 87.2 | 74.1 |
| *Mecopus* sp. | 78.5 | 79.3 | 74.0 | 69.7 | 68.9 | 83.4 | 75.5 |
| *Melanobaris laticollis* | 71.9 | 79.3 | 75.5 | 70.7 | 69.7 | 86.0 | 77.2 |
| *Melanterius* sp. | 80.7 | 78.7 | 77.4 | 71.9 | 69.5 | 90.8 | 76.4 |
| *Miarus* sp. | 80.1 | 78.2 | 76.6 | 71.1 | 69.4 | 89.4 | 75.0 |
| *Myllocerinus aurolineatus* | 79.6 | 78.4 | 74.8 | 68.9 | 67.8 | 87.7 | 75.1 |
| *Nanophyes marmoratus* | 76.6 | 79.1 | 75.0 | 69.9 | 67.6 | 87.7 | 75.7 |
| *Naupactus xanthographus* | 79.6 | 79.8 | 75.7 | 69.4 | 68.2 | 89.4 | 75.5 |
| *Niphades castanea* | 79.9 | 77.7 | 75.6 | 70.6 | 68.3 | 87.9 | 71.6 |
| *Orthotomicus laricis* | 75.4 | 76.1 | 71.2 | 66.3 | 66.2 | 80.9 | 73.6 |
| *Otiorhynchus rugosostriatus* | 69.4 | 76.8 | 73.7 | 69.3 | 67.7 | 84.2 | 75.7 |
| *Ouroporopterus* sp. | 78.6 | 78.1 | 75.8 | 70.9 | 68.5 | 87.9 | 74.1 |
| *Ceutorhynchus asper* | 75.2 | 78.1 | 73.9 | 69.4 | 67.7 | 84.5 | 75.6 |
| *Ceutorhynchus albosuturalis -* | | 78.8 | 75.4 | 70.6 | 67.7 | 88.0 | 73.3 |
| *Pachyrhinus yasumatsui* | 78.8 | 77.2 | 73.0 | 68.6 | 67.7 | 82.7 | 76.0 |
| *Pantoxystus rubricollis* | 78.6 | 79.0 | 76.2 | 70.1 | 68.8 | 89.8 | 71.8 |
| *Paroplapoderus tentator* | 76.4 | 73.0 | 71.9 | 66.8 | 65.7 | 83.1 | 68.9 |
| *Phloeosinus perlatus* | 77.1 | 74.2 | 68.2 | 64.9 | 66.0 | 73.8 | 77.7 |
| *Phloeotribus sp* | 82.0 | 80.1 | 77.6 | 72.2 | 69.7 | 90.8 | 74.4 |
| *Pimelocerus perforatus* | 79.1 | 78.7 | 74.1 | 68.2 | 67.6 | 86.3 | 74.4 |
| *Pissodes strobi* | 79.4 | 78.1 | 74.3 | 70.0 | 68.2 | 84.9 | 72.9 |
| *Pityogenes bidentatus* | 79.4 | 78.5 | 72.6 | 67.0 | 67.6 | 83.1 | 74.1 |
| *Platypus contaminatus* | 78.4 | 77.2 | 73.9 | 68.8 | 67.5 | 85.5 | 73.6 |
| *Platystomos albinus* | 79.4 | 76.4 | 73.4 | 67.5 | 68.4 | 84.3 | 69.1 |
| *Polygraphus poligraphus* | 74.9 | 72.1 | 68.9 | 63.9 | 65.7 | 77.1 | 74.7 |
| *Protapion interjectum* | 75.5 | 78.6 | 74.4 | 69.3 | 68.2 | 85.7 | 75.7 |
| *Protapion trifolii* | 79.8 | 79.7 | 75.6 | 70.3 | 69.0 | 87.5 | 77.1 |
| *Psylliodes chrysocephala* | 82.4 | 80.2 | 76.8 | 71.3 | 68.9 | 90.2 | 74.6 |
| *Rhinoncus* sp. | 78.5 | 77.5 | 74.4 | 69.6 | 68.1 | 85.5 | 69.5 |
| *Rhinostomus barbirostris* | 70.8 | 70.2 | 69.9 | 63.4 | 65.4 | 80.8 | 76.5 |
| *Rhopalapion longirostre* | 73.0 | 80.1 | 76.5 | 71.0 | 69.0 | 89.6 | 72.2 |
| *Rhynchitinae* sp. 1 | 77.4 | 76.1 | 71.8 | 67.3 | 66.3 | 82.0 | 74.5 |
| *Rhynchitinae* sp. 2 | 81.3 | 78.0 | 74.1 | 68.9 | 67.6 | 85.9 | 69.2 |
| *Rhynchitinae* sp. 3 | 77.0 | 74.7 | 68.6 | 64.5 | 66.2 | 75.0 | 71.9 |
| *Rhynchophorus ferrugineus* | 74.7 | 75.2 | 71.9 | 65.6 | 66.9 | 83.0 | 71.6 |
| *Rhythirrinus* sp. | 75.4 | 75.2 | 71.4 | 66.8 | 66.8 | 80.7 | 63.7 |
| *Scolytus schevyrewi* | 69.0 | 68.5 | 63.3 | 61.9 | 64.8 | 63.1 | 78.8 |
| *Sibinia fulva* | 81.0 | 81.2 | 78.9 | 74.1 | 70.4 | 92.2 | 65.4 |
| *Sipalinus gigas* | 70.8 | 69.8 | 65.0 | 61.3 | 65.1 | 68.6 | 75.5 |
| *Sitona callosus* | 78.2 | 79.4 | 75.5 | 69.6 | 67.6 | 89.4 | 75.6 |
| *Sitona lineatus* | 72.2 | 80.4 | 75.6 | 69.9 | 68.5 | 88.4 | 74.8 |
| *Sitophilus oryzae* | 78.9 | 78.3 | 74.8 | 71.1 | 68.1 | 85.3 | 76.4 |
| *Tomicus piniperda* | 80.4 | 79.6 | 76.4 | 71.1 | 68.5 | 89.6 | 70.7 |
| *Trigonopterus carinirostris* | 74.9 | 75.4 | 70.2 | 65.6 | 67.2 | 77.8 | 72.3 |
| *Tropiphorus elevatus* | 77.4 | 75.7 | 72.3 | 67.6 | 66.9 | 82.3 | 71.5 |
| *Tychius pusillus* | 79.7 | 77.8 | 76.0 | 70.1 | 68.4 | 89.6 | 75.9 |
| *Urodontus glabratus* | 76.6 | 75.2 | 72.5 | 66.5 | 68.8 | 82.2 | 72.6 |
| *Urodontus* sp. | 77.5 | 76.9 | 73.0 | 68.2 | 68.7 | 82.1 | 73.2 |

Note: PCG, protein coding genes; spaces represent missing values.

**Table S5. AT contents of 13 protein coding genes (“-” represent missing values)**

| **Species** | **ATP6** | **Cox1** | **ATP8** | **Cob** | **Cox3** | **Cox2** | **Nad1** | **Nad2** | **Nad3** | **Nad4** | **Nad4l** | **Nad5** | **Nad6** |
| --- | --- | --- | --- | --- | --- | --- | --- | --- | --- | --- | --- | --- | --- |
| *Acalyptus* sp. | 74.4 | 67.2 | 80.8 | 72.3 | 69.7 | 71.1 | 77.0 | 78.3 | 79.4 | 78.0 | 80.8 | 77.8 | 80.7 |
| *Aclees cribratus* | 74.5 | 68.1 | 82.1 | 70.0 | 69.2 | 72.2 | 74.9 | 75.9 | 78.2 | 78.5 | 83.7 | 78.5 | 80.0 |
| *Aegorhinus superciliosus* | 75.4 | 69.6 | 86.5 | 71.5 | 70.5 | 74.2 | 74.6 | 77.6 | 74.2 | 78.1 | 77.9 | 76.3 | 77.4 |
| *Alcidodes juglans* | 74.9 | 67.8 | 77.6 | 72.4 | 69.4 | 68.6 | 75.8 | 75.9 | 77.4 | 77.0 | 80.4 | 76.9 | 78.2 |
| *Ancyttalia* sp. | 75.9 | 69.9 | 80.1 | 72.4 | 72.1 | 73.8 | 76.1 | 78.6 | 78.8 | 78.1 | 82.0 | 77.9 | 84.1 |
| *Anisandrus dispar* | 69.9 | 65.9 | 78.9 | 68.2 | 68.5 | 68 | 72.7 | 75.0 | 74.0 | 72.4 | 74.5 | 73.3 | 75.7 |
| *Anthonomus eugenii* | 71.3 | 64.2 | 80.1 | 69.3 | 66.9 | 68.6 | 72.7 | 75.1 | 73.4 | 74.5 | 76.3 | 70.9 | 76.7 |
| *Anthonomus pomorum* | 71.7 | 68 | 78.9 | 70.7 | 70.8 | 71.9 | 73.1 | 76.6 | 76.6 | 74.1 | 78.7 | 73.5 | 80.2 |
| *Anthribidae* sp. | 71.0 | 64.6 | 80.5 | 69.0 | 68.6 | 70.8 | 76.8 | 76.5 | 74.0 | 76.4 | 78.1 | 77.0 | 78.8 |
| *Anthribidae* sp. 1 | 76.7 | 70.3 | 88.5 | 74.8 | 73.4 | 76.3 | 80.1 | 82.7 | 83.1 | 81.7 | 83.7 | 81.4 | 84.5 |
| *Anthribidae* sp. 8 | 71.3 | 66.5 | 79.5 | 68.8 | 70.1 | 71.6 | 74.0 | 77.9 | 76.8 | 78.2 | 77.8 | 76.9 | 81.0 |
| *Apoderinae* sp. 2 | 66.7 | 61.5 | 73.5 | 63.2 | 64.1 | 65.2 | 68.4 | - | 68.7 | 71.3 | 69.4 | 70.1 | 69.1 |
| *Apoderus coryli* | 70.1 | 62 | 80.7 | 66.6 | 67.7 | 66.4 | 71.4 | 66.8 | 73.6 | 73.2 | 70.1 | 72.2 | 72.5 |
| *Apoderus jekelii* | 71.0 | 63.8 | 78.2 | 67.5 | 68.4 | 68.4 | 71.7 | 68.5 | 73.3 | 74.8 | 70.5 | 73.2 | 71.9 |
| *Arachnobas tricolor* | 71.9 | 64.1 | 80.8 | 66.7 | 69.1 | 68.9 | 73.0 | 76.7 | 74.3 | 75.9 | 75.9 | 74.6 | 77.9 |
| *Attelabinae* sp. 1 | 64.3 | 61.7 | 75.6 | 64.0 | 66.2 | 64.8 | 67.4 | 69.7 | 69.2 | 69.8 | 69.5 | 69.4 | 70.3 |
| *Bagous* sp. | 65.5 | 62.8 | 76.7 | 65.1 | 64.8 | 66.4 | 72.2 | 70.2 | 71.8 | 72.7 | 70.2 | 71.8 | 76.3 |
| *Bangasternus* sp. | 74.8 | 69.8 | 82.1 | 73.6 | 72.8 | 73.3 | 77.8 | 79.1 | 78.8 | 78.5 | 83.0 | 78.3 | 82.6 |
| *Brachytemnus porcatus* | 73.1 | 68.4 | 78.2 | 69.4 | 69.5 | 70 | 74.4 | - | 74.0 | 74.9 | 79.6 | 73.9 | 77.7 |
| *Bradybatus kellneri* | 74.6 | 67.4 | 81.4 | 70.3 | 70.1 | 71.3 | 72.0 | 78.4 | 77.1 | 74.5 | 78.4 | 72.2 | 80.6 |
| *Camptorhinus* sp. | 77.6 | 69.6 | 83.0 | 73.2 | 72.7 | 72.9 | 78.2 | 80.4 | 79.9 | 79.7 | 81.3 | 80.3 | 81.7 |
| *Ceratopus* sp. | 72.4 | 69.7 | 83.3 | 71.9 | 69.8 | 72.5 | 75.7 | - | 72.6 | 78.3 | 80.3 | 76.8 | 80.9 |
| *Ceutorhynchus obstrictus* | 75.6 | 69.2 | 84.6 | 73.7 | 71.7 | 72.8 | 76.0 | - | 79.1 | 77.4 | 82.3 | 78.9 | 82.7 |
| *Chirotenon longimanus* | 72.2 | 67.8 | 78.2 | 69.6 | 69 | 71.8 | 75.3 | - | 75.7 | 78.0 | 81.2 | 78.0 | 77.0 |
| *Cionus griseus* | 78.1 | 68.5 | 82.7 | 73.0 | 72.9 | 72.7 | 76.9 | 81.1 | 78.8 | 77.3 | 82.1 | 77.8 | 81.5 |
| *Cionus olens* | 76.6 | 69.8 | 85.3 | 74.1 | 70.5 | 72 | 76.3 |  | 77.4 | 77.4 | 84.9 | 78.7 | 80.9 |
| *Cosmopolites sordidus* | 71.9 | 66 | 80.8 | 71.0 | 68.9 | 70.1 | 74.5 | 75.5 | 76.0 | 77.4 | 79.1 | 74.9 | 78.2 |
| *Cryphalus abietis* | 71.3 | 66.8 | 74.8 | 69.2 | 68.9 | 71.7 | 72.3 | 74.5 | 74.5 | 72.7 | 78.0 | 72.9 | 75.1 |
| *Curculio davidi* | 77.2 | 69.4 | 86.2 | 72.7 | 72.2 | 74.2 | 76.5 | 77.7 | 79.9 | 78.4 | 81.0 | 78.0 | 82.5 |
| *Curculio elephas* | 77.0 | 69.6 | 86.8 | 74.0 | 73.1 | 73.7 | 76.5 | 78.3 | 78.5 | 78.5 | 83.0 | 77.9 | 81.1 |
| *Cyllorhynchites cumulatus* | 78.1 | 64.1 | 84.3 | 66.4 | 68.6 | 65.8 | 70.0 | 75.7 | 73.2 | 73.1 | 74.7 | 73.7 | 76.3 |
| *Cyllorhynchites ursulus* | 68.1 | 62.4 | 78.2 | 64.7 | 64 | 65.2 | 68.4 | 72.8 | 72.6 | 74.4 | 75.0 | 73.4 | 75.0 |
| *Cyrtotrachelus buqueti* | 71.4 | 66.7 | 82.1 | 70.7 | 69.3 | 69.5 | 72.2 | 74.0 | 72.3 | 77.0 | 78.0 | 73.2 | 77.2 |
| *Cyrtotrachelus longimanus* | 71.3 | 67.9 | 80.1 | 69.8 | 69.8 | 69.8 | 72.5 | 73.8 | 73.2 | 77.2 | 78.4 | 73.5 | 77.4 |
| *Deporaus marginatus strain mangguoqieyexiangjia* | 72.7 | 68.4 | 80.1 | 70.7 | 70.1 | 69.9 | 77.1 | 76.2 | 77.7 | 78.5 | 78.8 | 77.8 | 82.5 |
| *Deporaus tristis* | 73.0 | 65 | 85.3 |  | 68.8 | 70.2 | - | 75.2 | 73.7 | 77.4 | 79.9 | 75.9 | 80.6 |
| *Doydirhynchus austriacus* | 75.4 | 71.2 | 84.0 | 73.1 | 72.4 | 74.7 | 78.6 | 76.1 | 80.2 | 80.3 | 84.0 | 79.5 | 85.1 |
| *Dryocoetes autographus* | 69.6 | 67.1 | 80.1 | 69.3 | 69.1 | 72.5 | 73.4 | - | 78.0 | 75.0 | 77.6 | 74.3 | 77.6 |
| *Dryophthoridae* sp. | 71.6 | 66.1 | 82.1 | 69.9 | 69.5 | 69.9 | 73.7 | 76.5 | 74.9 | 77.2 | 79.1 | 77.1 | 77.4 |
| *Echinodera andalusiensis* | 71.1 | 64.5 | 73.6 | 67.1 | 66.3 | 68.9 | 71.2 | 72.4 | 74.6 | 74.3 | 77.6 | 74.2 | 75.0 |
| *Elaeidobius kamerunicus* | 71.1 | 67.2 | 78.2 | 68.2 | 67.1 | 69.7 | 73.4 | 73.5 | 75.1 | 74.7 | 78.2 | 74.1 | 80.8 |
| *Eucryptorhynchus brandti* | 75.0 | 70 | 82.7 | 73.1 | 72.2 | 73.4 | 75.9 | 80.1 | 79.1 | 79.2 | 81.6 | 79.0 | 81.0 |
| *Euplatypus* sp. | 72.1 | 66 | 74.2 | 69.6 | 67.8 | 71.7 | 75.8 | 75.8 | 72.6 | 77.4 | 83.0 | 76.5 | 81.0 |
| *Gnathotrichus materiarius* | 63.4 | 58.8 | 66.7 | 60.8 | 61.4 | 61.4 | 65.7 | 64.5 | 68.1 | 65.0 | 68.0 | 64.2 | 67.5 |
| *Haplonyx* sp. | 71.6 | 64.7 | 78.9 | 69.4 | 66 | 68.3 | 72.2 | 76.3 | 75.1 | 75.3 | 77.9 | 73.7 | 78.2 |
| *Hylastes attenuatus* | 68.6 | 65.3 | 82.7 | 68.1 | 69.1 | 68.7 | 74.1 | 74.7 | 72.6 | 76.0 | 77.9 | 74.0 | 77.4 |
| *Hylesinus varius* | 77.1 | 72.1 | 87.2 | 76.0 | 76.4 | 75.4 | 79.6 | 81.6 | 82.2 | 81.0 | 79.7 | 80.2 | 84.7 |
| *Hylobitelus xiaoi* | 72.9 | 67.9 | 80.1 | 70.4 | 69.6 | 71.2 | 74.4 | 77.1 | 77.1 | 78.1 | 78.2 | 76.9 | 79.2 |
| *Hylobius abietis* | 73.9 | 68.9 | 79.4 | 69.9 | 69.2 | 71.5 | 73.0 | - | 79.7 | 76.3 | 77.6 | 76.5 | 80.0 |
| *Hypera postica* | 74.4 | 70.2 | 82.7 | 73.1 | 72.2 | 72.1 | 76.7 | 77.4 | 78.2 | 80.1 | 77.8 | 78.7 | 80.4 |
| *Hypothenemus* sp. | 64.3 | 61.7 | 75.6 | 65.7 | 65.6 | 66.2 | 71.5 | 71.9 | 68.4 | 71.5 | 75.7 | 71.1 | 70.6 |
| *Ips acuminatus* | 74.4 | 68.9 | 84.6 | 71.8 | 71.3 | 73.1 | 76.1 | 77.2 | 78.8 | 77.4 | 78.8 | 77.4 | 80.1 |
| *Kyklioacalles aubei* | 77.2 | 70.7 | 84.3 | 73.9 | 73.1 | 72.5 | 77.8 | - | 78.8 | 79.0 | 84.7 | 79.1 | 82.8 |
| *Laemosaccus* sp. | 75.6 | 69.1 | 79.5 | 73.9 | 73.9 | 73.7 | 77.4 | 80.7 | 79.1 | 78.2 | 81.6 | 79.8 | 79.9 |
| *Lepidapion squamigerum* | 74.6 | 68.3 | 84.0 | 73.0 | 69.8 | 73.5 | 76.2 | 80.8 | 79.1 | 78.8 | 80.7 | 79.2 | 81.8 |
| *Leptomias* sp. | 72.4 | 64.4 | 76.3 | 67.9 | 68.4 | 69.9 | 73.1 | 74.5 | 71.8 | 76.7 | 75.7 | 75.4 | 76.4 |
| *Lixus subtilis* | 72.6 | 68 | 84.6 | 71.6 | 69.7 | 72.5 | 75.5 | 77.8 | 77.1 | 79.4 | 81.4 | 78.3 | 82.4 |
| *Mecopus* sp. | 73.9 | 67.6 | 84.6 | 70.8 | 69.1 | 72.4 | 73.0 | 77.8 | 78.5 | 75.7 | 82.7 | 77.2 | 78.8 |
| *Melanobaris laticollis* | 74.6 | 69.1 | 86.5 | 71.0 | 69.6 | 72.5 | 76.4 | - | 78.2 | 77.4 | 84.2 | 79.1 | 81.6 |
| *Melanterius* sp. | 78.1 | 70.7 | 84.6 | 75.7 | 74 | 72.8 | 77.0 | 81.2 | 80.2 | 80.2 | 82.0 | 80.2 | 81.9 |
| *Miarus* sp. | 77.0 | 70 | 87.2 | 74.2 | 74.3 | 75.1 | 76.4 | 81.5 | 79.7 | 79.0 | 78.6 | 77.6 | 81.7 |
| *Myllocerinus aurolineatus* | 74.4 | 69.4 | 80.1 | 72.3 | 71.1 | 72.1 | 77.4 | 77.4 | 77.7 | 78.4 | 76.0 | 75.6 | 81.1 |
| *Nanophyes* | 74.1 | 67 | 80.8 | 71.8 | 70.1 | 71.2 | 77.3 | - | 76.3 | 79.4 | 82.3 | 78.6 | 81.0 |
| *Naupactus xanthographus* | 76.3 | 69.4 | 83.3 | 73.3 | 70.5 | 70.7 | 76.9 | 79.1 | 77.4 | 79.9 | 78.5 | 78.0 | 81.3 |
| *Niphades castanea* | 76.3 | 69.1 | 82.7 | 70.7 | 71.4 | 73.7 | 76.2 | 80.0 | 76.8 | 78.4 | 82.0 | 77.5 | 84.2 |
| *Orthotomicus laricis* | 68.4 | 65.7 | 75.2 | 68.7 | 66.5 | 68.6 | 73.2 | 73.7 | 74.3 | 74.1 | 78.2 | 73.4 | 76.6 |
| *Otiorhynchus rugosostriatus* | 75.0 | 69.1 | 76.9 | 72.2 | 68.2 | 70.8 | 74.4 | - | 74.6 | 77.5 | 76.4 | 74.4 | 79.2 |
| *Ouroporopterus sp* | 75.4 | 68.9 | 82.1 | 72.9 | 70.1 | 74 | 76.3 | 80.2 | 81.4 | 77.8 | 80.3 | 78.8 | 82.3 |
| *Ceutorhynchus asper* | 74.3 | 68.4 | 83.3 | 71.3 | 70.5 | 71.8 | 74.3 | 75.8 | 77.7 | 76.5 | 79.0 | 74.5 | 82.2 |
| *Ceutorhynchus albosuturalis* | 76.6 | 69.1 | - | - | 71.7 | 73.3 |  | 77.5 | 79.9 | 77.2 | 82.1 | 78.0 | - |
| *Pachyrhinus yasumatsui* | 72.1 | 67.6 | 85.9 | 69.8 | 69.2 | 70.6 | 74.8 | 73.7 | 73.7 | 78.0 | 79.2 | 73.8 | 79.0 |
| *Pantoxystus rubricollis* | 75.3 | 69.3 | 84.6 | 71.6 | 71.6 | 72.5 | 76.4 | 82.0 | 79.7 | 79.8 | 82.8 | 78.8 | 83.8 |
| *Paroplapoderus tentator* | 72.5 | 67 | 83.3 | 68.5 | 70.2 | 68 | 74.9 | 71.1 | 73.9 | 75.8 | 72.6 | 74.9 | 73.3 |
| *Phloeosinus perlatus* | 67.4 | 63.3 | 76.9 | 65.4 | 64.9 | 66.7 | 70.8 | 71.0 | 68.4 | 71.5 | 72.9 | 68.3 | 75.0 |
| *Phloeotribus* sp. | 76.6 | 71.2 | 75.8 | 73.9 | 71.3 | 75.2 | 78.6 | 80.9 | 81.9 | 80.8 | 83.0 | 80.4 | 84.2 |
| *Pimelocerus perforatus* | 72.9 | 68.3 | 85.9 | 71.5 | 70.1 | 69.8 | 75.2 | 77.6 | 76.8 | 76.6 | 79.6 | 76.5 | 79.0 |
| *Pissodes strobi* | 74.0 | 67.4 | 82.1 | 70.5 | 69.6 | 73.5 | 74.6 | 77.8 | 78.0 | 77.6 | 80.3 | 77.9 | 79.2 |
| *Pityogenes bidentatus* | 70.5 | 65.6 | 75.0 | 69.3 | 67.6 | 70.9 | 74.5 | 75.9 | 73.2 | 75.5 | 76.4 | 76.2 | 78.8 |
| *Platypus contaminatus* | 72.4 | 68 | 79.9 | 70.6 | 69.7 | 70.8 | 75.4 | 76.5 | 71.5 | 77.4 | 79.9 | 77.2 | 81.0 |
| *Platystomos albinus* | 71.9 | 65.4 | 79.0 | 68.6 | 70.3 | 70.8 | 75.2 | 76.3 | 77.7 | 77.9 | 80.6 | 77.1 | 77.8 |
| *Polygraphus poligraphus* | 65.9 | 63.3 | 76.1 | 66.1 | 66.5 | 68.1 | 72.8 | 71.9 | 69.8 | 71.7 | 72.8 | 69.7 | 73.4 |
| *Protapion interjectum* | 72.2 | 68.7 | 74.2 | - | 69.4 | 72.9 | - | 79.3 | 76.6 | 76.1 | - | 77.9 | - |
| *Protapion trifolii* | 73.7 | 69 | 83.3 | 70.4 | 70.8 | 74.5 | 76.5 | 79.7 | 79.0 | 77.4 | 80.0 | 79.3 | 83.3 |
| *Rhinoncus sp* | 74.9 | 68 | 88.5 | 71.8 | 71 | 71.8 | 75.2 | 77.3 | 76.0 | 76.4 | 78.5 | 77.0 | 82.1 |
| *Rhinostomus barbirostris* | 68.6 | 64.9 | 77.6 | 68.5 | 67.3 | 66 | 71.6 | 71.0 | 71.0 | 73.3 | 75.8 | 71.9 | 72.6 |
| *Rhopalapion longirostre* | 77.0 | 70.7 | 75.0 | 72.6 | 72 | 74.6 | 77.5 | - | 80.5 | 79.9 | 80.2 | 79.6 | 83.4 |
| *Rhynchitinae* sp. 1 | 70.8 | 64.3 | 86.5 | 69.4 | 66.5 | 68.9 | 73.6 | 73.7 | 75.7 | 76.0 | 75.7 | 75.5 | 77.5 |
| *Rhynchitinae* sp. 2 | 73.3 | 65.9 | 75.6 | 72.5 | 69.7 | 70.2 | 76.5 | 75.4 | 78.8 | 77.9 | 77.4 | 76.5 | 82.2 |
| *Rhynchitinae* sp. 3 | 66.8 | 63.3 | 86.2 | 64.3 | 63.5 | 67.9 | 70.4 | 69.7 | 68.1 | 72.0 | 75.0 | 72.2 | 73.6 |
| *Rhynchophorus ferrugineus* | 71.3 | 65.7 | 74.4 | 68.4 | 67.6 | 68.9 | 71.7 | 74.8 | 73.4 | 75.8 | 75.9 | 74.4 | 78.6 |
| *Rhythirrinus* sp. | 71.4 | 65.7 | 84.6 | 68.8 | 67.9 | 68.3 | 72.2 | 73.5 | 68.6 | 75.4 | 72.9 | 74.8 | 76.6 |
| *Scolytus schevyrewi* | 62.5 | 57.9 | 77.6 | 61.1 | 61.2 | 61.6 | 65.4 | 64.9 | 65.5 | 67.3 | 69.4 | 63.8 | 65.1 |
| *Sibinia fulva* | 79.5 | 72.4 | 67.3 | 75.7 | 76 | 74.5 | 78.9 | 82.4 | 80.6 | 82.0 | 85.9 | 81.5 | 83.7 |
| *Sipalinus gigas* | 62.4 | 59.7 | 88.0 | 61.8 | 62.9 | 66.7 | 66.6 | 67.4 | 63.6 | 66.5 | 72.6 | 67.6 | 69.2 |
| *Sitona callosus* | 75.0 | 68.6 | 67.3 | 72.0 | 72.8 | 74.5 | 75.7 | 78.7 | 79.4 | 79.8 | 78.8 | 77.2 | 79.9 |
| *Sitona lineatus* | 75.7 | 70.4 | 85.3 | 71.8 | 72.2 | 72.1 | 76.2 | - | 77.4 | 78.7 | 79.5 | 77.2 | 81.7 |
| *Sitophilus oryzae* | 74.0 | 67.9 | 86.3 | 72.1 | 70.7 | 72.7 | 76.7 | 77.3 | 76.6 | 78.5 | 81.6 | 76.9 | 81.1 |
| *Tomicus piniperda* | 74.8 | 70.4 | 80.1 | 73.4 | 72 | 73.2 | 76.5 | 79.3 | 77.7 | 79.5 | 80.2 | 79.7 | 83.4 |
| *Trigonopterus carinirostris* | 68.6 | 65.2 | 84.9 | 69.3 | 65.5 | 66.8 | 73.1 | 71.4 | 74.0 | 73.5 | 74.5 | 72.0 | 72.4 |
| *Tropiphorus elevatus* | 71.3 | 66.1 | 73.9 | 69.3 | 67.9 | 71.3 | 73.6 | 75.1 | 72.9 | 77.0 | 75.0 | 73.3 | 79.5 |
| *Tychius pusillus* | 75.4 | 68.3 | 76.9 | 73.9 | 71.9 | 74.9 | 76.3 | 79.8 | 81.1 | 79.1 | 78.7 | 78.6 | 81.9 |
| *Urodontus glabratus* | 71.0 | 66.8 | 81.8 | 69.6 | 67.3 | 68.4 | 73.7 | - | 75.7 | 76.9 | 75.1 | 75.2 | 76.9 |
| *Urodontus* sp. | 71.9 | 66.9 | 77.6 | 69.8 | 69.9 | 69.9 | 74.1 | - | 75.7 | 76.6 | 77.5 | 74.8 | 76.8 |
| *Xylosandrus crassiusculus* | 72.2 | 67.1 | 80.8 | 69.9 | 71.3 | 71.8 | 74.9 | 74.8 | 74.0 | 75.9 | 75.2 | 75.0 | 74.9 |

**Table S6. Comparison of nucleotide compositions of mitochondrial genomes of species in Curculionoidea**

| **Species** | **AT%** | **AT skew** | **GC%** | **GC skew** |
| --- | --- | --- | --- | --- |
| *Rhynchitinae* sp. 1 | 71.8 | - .122 | 28.2 | - .030 |
| *Ancyttalia* sp. | 75.8 | - .127 | 24.2 | - .039 |
| *Hylobius abietis* | 73.9 | - .147 | 26.1 | - .035 |
| *Rhinostomus barbirostris* | 69.9 | - .132 | 30.1 | - .052 |
| *Kyklioacalles aubei* | 76.9 | - .148 | 23.1 | - .021 |
| *Melanobaris laticollis* | 75.5 | - .158 | 24.5 | - .022 |
| *Protapion interjectum* | 74.4 | - .133 | 25.6 | - .050 |
| *Otiorhynchus rugosostriatus* | 73.7 | - .144 | 26.3 | - .020 |
| *Doydirhynchus austriacus* | 77.3 | - .140 | 22.7 | .034 |
| *Acanthoscelides obtectus* | 74.2 | - .153 | 25.8 | - .025 |
| *Tropiphorus elevatus* | 72.3 | - .140 | 27.7 | - .086 |
| *Rhinoncus* sp. | 74.4 | - .139 | 25.6 | - .032 |
| *Naupactus xanthographus* | 75.7 | - .148 | 24.3 | - .021 |
| *Elaeidobius kamerunicus* | 72.0 | - .128 | 28.0 | - .068 |
| *Sitona callosus* | 75.5 | - .121 | 24.5 | - .018 |
| *Cionus olens* | 75.9 | - .124 | 24.1 | - .002 |
| *Platystomos albinus* | 73.4 | - .165 | 26.6 | - .049 |
| *Xylosandrus crassiusculus* | 72.9 | - .120 | 27.1 | - .060 |
| *Lixus subtilis* | 75.0 | - .135 | 25.0 | - .037 |
| *Apoderus coryli* | 69.1 | - .123 | 30.9 | - .037 |
| *Platypus contaminatus* | 73.9 | - .165 | 26.1 | - .030 |
| *Hylobitelus xiaoi* | 74.0 | - .141 | 26.0 | - .069 |
| *Pissodes strobi* | 74.3 | - .137 | 25.7 | - .052 |
| *Tychius pusillus* | 76.0 | - .123 | 24.0 | - .024 |
| *Alcidodes juglans* | 74.0 | - .120 | 26.0 | - .032 |
| *Psylliodes chrysocephala* | 76.8 | - .132 | 23.2 | - .022 |
| *Dryocoetes autographus* | 72.6 | - .140 | 27.4 | - .018 |
| *Anthonomus pomorum* | 73.0 | - .132 | 27.0 | - .057 |
| *Leptomias* sp. | 71.9 | - .135 | 28.1 | - .089 |
| *Bagous* sp. | 68.8 | - .153 | 31.2 | - .056 |
| *Hylesinus varius* | 78.4 | - .128 | 21.6 | - .018 |
| *Sitona lineatus* | 75.6 | - .138 | 24.4 | .003 |
| *Gnathotrichus materiarius* | 63.3 | - .099 | 36.7 | - .083 |
| *Anisandrus dispar* | 71.1 | - .115 | 28.9 | - .044 |
| *Rhythirrinus* sp. | 71.4 | - .133 | 28.6 | - .063 |
| *Niphades castanea* | 75.6 | - .147 | 24.4 | - .021 |
| *Curculio elephas* | 76.1 | - .142 | 23.9 | - .053 |
| *Cyllorhynchites cumulatus* | 71.0 | - .127 | 29.0 | - .026 |
| *Bangasternus* sp. | 76.2 | - .143 | 23.8 | - .023 |
| *Urodontus* sp. | 73.0 | - .192 | 27.0 | - .003 |
| *Phloeosinus perlatus* | 68.2 | - .111 | 31.8 | - .097 |
| *Cosmopolites sordidus* | 73.0 | - .135 | 27.0 | - .078 |
| *Orthotomicus laricis* | 71.2 | - .168 | 28.8 | - .022 |
| *Cyrtotrachelus longimanus* | 72.5 | - .134 | 27.5 | - .076 |
| *Scolytus schevyrewi* | 63.3 | - .106 | 36.7 | - .088 |
| *Chirotenon longimanus* | 74.1 | - .181 | 25.9 | - .035 |
| *Hypothenemus* sp. | 68.3 | - .081 | 31.7 | - .080 |
| *Apoderinae* sp. 2 | 66.9 | - .140 | 33.1 | - .025 |
| *Camptorhinus* sp. | 76.8 | - .157 | 23.2 | - .052 |
| *Hypera postica* | 75.8 | - .147 | 24.2 | .005 |
| *Trigonopterus carinirostris* | 70.2 | - .137 | 29.8 | - .078 |
| *Anthribidae* sp. 8 | 73.7 | - .144 | 26.3 | - .047 |
| *Nanophyes marmoratus* | 75.0 | - .137 | 25.0 | - .018 |
| *Tomicus piniperda* | 76.4 | - .140 | 23.6 | - .014 |
| *Anthribidae* sp. 1 | 78.4 | - .159 | 21.6 | - .020 |
| *Cyllorhynchites ursulus* | 69.3 | - .129 | 30.7 | - .042 |
| *Laemosaccus* sp. | 76.4 | - .144 | 23.6 | - .085 |
| *Acalyptus* sp. | 74.9 | - .125 | 25.1 | - .043 |
| *Callosobruchus maculatus* | 73.3 | - .140 | 26.7 | - .041 |
| *Mecopus* sp. | 74.0 | - .139 | 26.0 | - .057 |
| *Sipalinus gigas* | 65.0 | - .138 | 35.0 | - .084 |
| *Cionus griseus* | 75.9 | - .117 | 24.1 | - .017 |
| *Rhynchitinae* sp. 2 | 74.1 | - .135 | 25.9 | - .031 |
| *Eucryptorhynchus brandti* | 76.2 | - .157 | 23.8 | - .046 |
| *Euplatypus* sp. | 73.3 | - .146 | 26.7 | - .029 |
| *Cryphalus abietis* | 71.6 | - .151 | 28.4 | - .009 |
| *Phloeotribus sp.* | 77.6 | - .139 | 22.4 | - .028 |
| *Pantoxystus rubricollis* | 76.2 | - .110 | 23.8 | - .038 |
| *Curculio davidi* | 75.9 | - .146 | 24.1 | - .049 |
| *Miarus* sp. | 76.6 | - .126 | 23.4 | - .008 |
| *Ceutorhynchus albosuturalis* | 75.4 | - .116 | 24.6 | - .022 |
| *Pachyrhinus yasumatsui* | 73.0 | - .135 | 27.0 | - .049 |
| *Sibinia fulva* | 78.9 | - .132 | 21.1 | - .029 |
| *Deporaus tristis* | 73.4 | - .129 | 26.6 | - .033 |
| *Apoderus jekelii* | 70.2 | - .127 | 29.8 | - .049 |
| *Lepidapion squamigerum* | 75.8 | - .130 | 24.2 | - .009 |
| *Cyrtotrachelus buqueti* | 72.2 | - .126 | 27.8 | - .076 |
| *Attelabinae* sp. 1 | 67.0 | - .111 | 33.0 | - .046 |
| *Pimelocerus perforatus* | 74.1 | - .140 | 25.9 | - .041 |
| *Urodontus glabratus* | 72.5 | - .191 | 27.5 | - .022 |
| *Dryophthoridae* sp. | 73.2 | - .159 | 26.8 | - .064 |
| *Ceutorhynchus asper* | 73.9 | - .125 | 26.1 | - .037 |
| *Rhopalapion longirostre* | 76.5 | - .148 | 23.5 | .001 |
| *Haplonyx* sp. | 71.7 | - .119 | 28.3 | - .065 |
| *Sitophilus oryzae* | 74.8 | - .134 | 25.2 | - .093 |
| *Protapion trifolii* | 75.6 | - .146 | 24.4 | - .012 |
| *Hylastes attenuatus* | 71.8 | - .145 | 28.2 | - .064 |
| *Anthonomus eugenii* | 71.0 | - .134 | 29.0 | - .085 |
| *Melanterius* sp. | 77.4 | - .147 | 22.6 | - .039 |
| *Arachnobas tricolor* | 71.9 | - .164 | 28.1 | - .049 |
| *Myllocerinus aurolineatus* | 74.8 | - .165 | 25.2 | - .014 |
| *Ceratopus* sp. | 74.7 | - .147 | 25.3 | - .026 |
| *Ceutorhynchus obstrictus* | 76.0 | - .139 | 24.0 | - .005 |
| *Aclees cribratus* | 74.6 | - .143 | 25.4 | - .060 |
| *Paroplapoderus tentator* | 71.9 | - .124 | 28.1 | - .033 |
| *Echinodera andalusiensis* | 70.8 | - .124 | 29.2 | - .099 |
| *Ouroporopterus* sp. | 75.8 | - .146 | 24.2 | - .015 |
| *Deporaus marginatus strain mangguoqieyexiangjia* | 74.6 | - .146 | 25.4 | - .023 |
| *Rhynchitinae* sp. 3 | 68.6 | - .135 | 31.4 | - .046 |
| *Aegorhinus superciliosus* | 74.6 | - .120 | 25.4 | - .076 |
| *Ips acuminatus* | 74.9 | - .153 | 25.1 | - .036 |
| *Brachytemnus porcatus* | 72.8 | - .162 | 27.2 | - .035 |
| *Anthribidae* sp. | 73.0 | - .153 | 27.0 | - .065 |
| *Polygraphus poligraphus* | 68.9 | - .167 | 31.1 | - .100 |
| *Rhynchophorus ferrugineus* | 71.9 | - .165 | 28.1 | - .075 |
| *Pityogenes bidentatus* | 72.6 | - .154 | 27.4 | - .010 |
| *Bradybatus kellneri* | 72.9 | - .132 | 27.1 | - .067 |

**Table S7. Codon usage of protein coding genes for mitogenomes of Curculionoidea.**

| **Genes** | **Start codon** | | | | | | | | | | | | | | | **Stop codon** | | |
| --- | --- | --- | --- | --- | --- | --- | --- | --- | --- | --- | --- | --- | --- | --- | --- | --- | --- | --- |
|  | ATT | ATA | ATG | ATC | TTG | TCA | TTA | TTT | TCG | CCG | CGA | GAA | AAT | CTC | CAA | TAG | TAA | T-- |
| Nad2 | 80 | 7 | 5 |  |  |  |  |  |  |  |  |  |  |  |  | 10 | 82 |  |
| Cox1 | 89 | 2 | 1 | 1 | 4 |  | 2 |  | 5 | 1 | 1 | 1 | 1 |  |  |  |  | 3 |
| Cox2 | 44 | 40 | 9 | 13 | 1 |  |  |  |  |  |  |  |  | 1 |  | 4 | 98 | 6 |
| ATP8 | 79 | 11 |  | 18 |  |  |  |  |  |  |  |  |  |  |  | 52 | 56 |  |
| ATP6 |  | 4 | 104 |  |  |  |  |  |  |  |  |  |  |  |  | 2 | 106 |  |
| Cox3 | 3 | 1 | 103 |  |  |  |  |  |  |  |  |  |  |  | 1 | 7 | 89 | 12 |
| Nad3 | 62 | 41 | 5 |  |  |  |  |  |  |  |  |  |  |  |  | 47 | 60 | 1 |
| Nad5 | 101 | 2 | 4 |  |  | 1 |  |  |  |  |  |  |  |  |  | 34 | 65 | 9 |
| Nad4 | 6 | 8 | 90 |  | 2 |  | 1 | 1 |  |  |  |  |  |  |  | 18 | 52 | 38 |
| Nad4L | 1 | 3 | 103 |  |  |  |  |  |  |  |  |  |  |  |  | 18 | 89 |  |
| Nad6 | 93 | 11 | 2 |  |  |  |  |  |  |  |  |  |  |  |  | 1 | 105 |  |
| Cytb | 1 |  | 104 |  |  |  |  |  |  |  |  |  |  |  |  | 60 | 45 |  |
| Nad1 | 30 | 58 | 16 |  | 1 |  |  |  |  |  |  |  |  |  |  | 79 | 26 |  |

**Table S8. Relative synonymous codon usage (RSCU) for mitogenomes of Curculionoidea**

| **Amino acids** | **RSCU** | | | | | | **Anticodon** | | | | | |
| --- | --- | --- | --- | --- | --- | --- | --- | --- | --- | --- | --- | --- |
| **Phe** | 1.57 | 0.43 |  |  |  |  | **UUU(F)** | **UUC(F)** |  |  |  |  |
| **Leu** | 2.63 | 0.89 | 1.05 | 0.38 | 0.74 | 0.3 | **UUA(L)** | **UUG(L)** | **CUU(L)** | **CUC(L)** | **CUA(L)** | **CUG(L)** |
| **Ile** | 1.6 | 0.4 | 1.47 |  |  |  | **AUU(I)** | **AUC(I)** | **AUA(I)** |  |  |  |
| **Met** | 0.53 |  |  |  |  |  | **AUG(M)** |  |  |  |  |  |
| **Val** | 1.77 | 0.47 | 1.27 | 0.49 |  |  | **GUU(V)** | **GUC(V)** | **GUA(V)** | **GUG(V)** |  |  |
| **Ser** | 1.71 | 0.8 | 1.43 | 0.34 | 0.94 | 0.68 | **UCU(S)** | **UCC(S)** | **UCA(S)** | **UCG(S)** | **AGU(S)** | **AGC(S)** |
| **Pro** | 1.58 | 1.06 | 1.08 | 0.28 |  |  | **CCU(P)** | **CCC(P)** | **CCA(P)** | **CCG(P)** |  |  |
| **Thr** | 1.58 | 0.93 | 1.17 | 0.32 |  |  | **ACU(T)** | **ACC(T)** | **ACA(T)** | **ACG(T)** |  |  |
| **Ala** | 1.8 | 0.83 | 1.14 | 0.23 |  |  | **GCU(A)** | **GCC(A)** | **GCA(A)** | **GCG(A)** |  |  |
| **Tyr** | 1.56 | 0.44 |  |  |  |  | **UAU(Y)** | **UAC(Y)** |  |  |  |  |
| **Gly** | 1.02 | 0.5 | 1.43 | 1.05 |  |  | **GGU(G)** | **GGC(G)** | **GGA(G)** | **GGG(G)** |  |  |
| **His** | 1.45 | 0.55 |  |  |  |  | **CAU(H)** | **CAC(H)** |  |  |  |  |
| **Gln** | 1.38 | 0.62 |  |  |  |  | **CAA(Q)** | **CAG(Q)** |  |  |  |  |
| **Asn** | 1.56 | 0.44 |  |  |  |  | **AAU(N)** | **AAC(N)** |  |  |  |  |
| **Lys** | 1.46 | 0.54 |  |  |  |  | **AAA(K)** | **AAG(K)** |  |  |  |  |
| **Asp** | 1.5 | 0.5 |  |  |  |  | **GAU(D)** | **GAC(D)** |  |  |  |  |
| **Glu** | 1.31 | 0.69 |  |  |  |  | **GAA(E)** | **GAG(E)** |  |  |  |  |
| **Cys** | 1.26 | 0.74 |  |  |  |  | **UGU(C)** | **UGC(C)** |  |  |  |  |
| **Trp** | 0.75 |  |  |  |  |  | **UGG(W)** |  |  |  |  |  |
| **Arg** | 1.09 | 0.53 | 1.5 | 0.89 | 1.19 | 0.92 | **CGU(R)** | **CGC(R)** | **CGA(R)** | **CGG(R)** | **AGA(R)** | **AGG(R)** |
| **End** | 1.35 | 0.65 | 1.25 |  |  |  | **UAA(*)** | **UAG(*)** | **UGA(*)** |  |  |  |

Note: Codons with RSCU values above 1 represent strong bias for the corresponding codons, and are referred to as frequent codons. Codons with RSCU values below one are less-frequent codons.

**Table S9. Non-synonymous (Ka) and synonymous (Ks) substitution ratios of 13 proteins**

| **Proteins** | **Ka** | **Ks** | **Ka/Ks** |
| --- | --- | --- | --- |
| Cox1 | 0.42409 | 0.15708 | 2.6998345 |
| Nad5 | 0.28083 | 0.30837 | 0.9106917 |
| Nad4 | 0.22943 | 0.27207 | 0.8432756 |
| Nad2 | 0.29846 | 0.43838 | 0.6808249 |
| Nad1 | 0.23286 | 0.31776 | 0.7328172 |
| Cob | 0.28848 | 0.23866 | 1.2087488 |
| Nad6 | 0.57495 | 0.26231 | 2.1918722 |
| Nad4l | 0.2195 | 0.50755 | 0.4324697 |
| Cox3 | 0.16368 | 0.6079 | 0.2692548 |
| ATP8 | 0.40374 | 0.52157 | 0.7740859 |
| ATP6 | 0.4622 | 0.57294 | 0.8067162 |
| Cox2 | 0.13324 | 0.61098 | 0.2180759 |
| Nad3 | 0.33867 | 0.15729 | 2.1531566 |

**Table S10. The mean pairwise genetic distance and nucleotide diversity of 13 proteins.**

| **Proteins** | **Mean genetic distance** | **Pi** |
| --- | --- | --- |
| ATP6 | 0.501 | 0.28321 |
| ATP8 | 0.699 | 0.32343 |
| Cob | 0.39 | 0.24977 |
| Cox1 | 0.315 | 0.21721 |
| Cox2 | 0.367 | 0.23608 |
| Cox3 | 0.4 | 0.26308 |
| Nad1 | 0.407 | 0.25092 |
| Nad2 | 0.78 | 0.33145 |
| Nad3 | 0.575 | 0.30172 |
| Nad4 | 0.471 | 0.26715 |
| Nad4l | 0.637 | 0.30242 |
| Nad5 | 0.574 | 0.29221 |
| Nad6 | 0.805 | 0.33522 |

**Table S11 Host plant associations of *Ceutorhynchus* species**

| **Orginasim** | **Larval feeding site** | **Hosts and feeding habits** | **References** |
| --- | --- | --- | --- |
| *Ceutorhynchus napi* | Petiole or stem | Brassicaceae, oligophagous | Balalaikins, 2012; Buhr, 1964; Compte, 1981; Rheinheimer and Hassler, 2010; Delbol, 2008; |
| *Ceutorhynchus carinatus* | Petiole or the axis of the inflorescence | Brassicaceae, oligophagous | Nikolai et al., 2018, Rheinheimer and Hassler, 2010; Gültekin 2014; Avgın and Colonnell, 2011; Delbol, 2008; |
| *Ceutorhynchus rapae* | Petiole or stem | Brassicaceae, oligophagous | Rheinheimer and Hassler, 2010; Delbol, 2008; Heijerman, 1993 |
| *Ceutorhynchus sophiae* | Petiole and stem | Brassicaceae, oligophagous | Nikolai et al., 2018; Balalaikins, 2012; Dieckmann, 1972 |
| *Ceutorhynchus ignitus* | Stem | Brassicaceae, oligophagous | Nikolai et al., 2018; Rheinheimer and Hassler, 2010; Delbol, 2008; Delbol, 2013 |
| *Ceutorhynchus pervicax* | Stem | Brassicaceae, monophagous | Nikolai et al., 2018; Rheinheimer and Hassler, 2010; Delbol, 2008; Delbol, 2013 |
| *Ceutorhynchus barbarae* | Stem | Brassicaceae, oligophagous | Nikolai et al., 2018; Rheinheimer and Hassler, 2010; Delbol, 2008; Delbol, 2013; Dieckmann, 1972a; Germann, 2011 |
| *Ceutorhynchus typhae* | Fruits | Brassicaceae, oligophagous | Nikolai et al., 2018; Rheinheimer and Hassler, 2010; Dieckmann, 1972; Gebiola et al., 2015 |
| *Ceutorhynchus cakilis* | Fruits | Brassicaceae, oligophagous | Dieckmann, 1972; Benedikt et al., 2010; Delbol, 2008; Delbol, 2013 |
| *Ceutorhynchus pulvinatus* |  | Brassicaceae, oligophagous | Nikolai et al., 2018; Delbol, 2008; Delbol, 2013 |
| *Ceutorhynchus niyazii* | Fruits | Brassicaceae, narrowly monophahous | Benedikt et al., 2010; Nikolai et al., 2018; Rheinheimer and Hassler, 2010 |
| *Ceutorhynchus moraviensis* | - | Brassicaceae, narrowly monophagous | Benedikt et al., 2010 |
| *Ceutorhynchus sisymbrii* |  | Brassicaceae, monophagous | Dieckmann, 1972; Nikolai et al., 2018 |
| *Ceutorhynchus rhenanus* | Fruits | Brassicaceae, oligofaag | Delbol, 2008; Delbol, 2013; Rheinheimer and Hassler, 2010; Nikolai et al., 2018 |
| *Ceutorhynchus pumilio* | Siliquaes | Brassicaceae, monophagous | Nikolai et al., 2018; Rheinheimer and Hassler, 2010; Behne, 1987; Delbol, 2008; Delbol, 2013 |
| *Ceutorhynchus pyrrhorhynchus* | Fruits | Probably the larvae live in the fruits | Nikolai et al., 2018; Rheinheimer and Hassler, 2010; Delbol, 2008; Delbol, 2013; Aydın and Hacet, 2016 |
| *Ceutorhynchus arator* |  | Brassicaceae, oligophagous | Rheinheimer and Hassler, 2010 |
| *Ceutorhynchus inaffectatus* | Siliques and seeds | Brassicaceae, oligophagous | Rheinheimer and Hassler, 2010; Delbol, 2008; Delbol, 2013; Abbazzi et al., 2016 |
| *Ceutorhynchus aeneicollis* | Stem | Brassicaceae, oligophagous | Dieckmann, 1972; Rheinheimer and Hassler, 2010; Nikolai et al., 2018 |
| *Ceutorhynchus chlorophanus* |  | Brassicaceae, monophagous | Rheinheimer and Hassler, 2010 |
| *Ceutorhynchus hampei* | Fruits | Brassicaceae, monophagous | Delbol, 2008; Delbol, 2013; Rheinheimer and Hassler, 2010; Dieckmann, 1972 |
| *Ceutorhynchus libertorum* |  | Brassicaceae, monophagous | Rheinheimer and Hassler, 2010 |
| *Ceutorhynchus liliputanus* |  | Brassicaceae, monophagous | Rheinheimer and Hassler, 2010 |
| *Ceutorhynchus parvulus* | Siliques | Brassicaceae, oligophagous | Delbol, 2008; Delbol, 2013; Nikolai et al., 2018; Rheinheimer and Hassler, 2010 |
| *Ceutorhynchus turbatus* | Siliques and seeds | Brassicaceae, monophagous | Berg C van et al., 1999; Delbol, 2008; Delbol, 2013; Nikolai et al., 2018 |
| *Ceutorhynchus gallorhenanus* | Flowers | Brassicaceae, broadly oligophagous | Delbol, 2008a; Delbol, 2013; Rheinheimer and Hassler, 2010; Nikolai et al., 2018 |
| *Ceutorhynchus obstrictus* | Siliquaes and seeds | Brassicaceae, broadly oligophagous | Colonnelli, 1990a; Delbol, 2008; Delbol, 2013; Rheinheimer and Hassler, 2010; Nikolai et al., 2018 |
| *Ceutorhynchus lukesi* | - | Brassicaceae, narrowly monophagous | Rheinheimer and Hassler, 2010 |
| *Ceutorhynchus pallidactylus* | Petiole or stem | Brassicaceae, Resedaceae, Tropaeolaceae | Colonnelli, 1990; Compte, 1981; Rheinheimer and Hassler, 2010 |
| *Ceutorhynchus sulcicollis* | Stem | Brassicaceae, oligophagous | Delbol, 2008; Delbol, 2013; Compte, 1981; Nikolai et al., 2018 |
| *Ceutorhynchus picitarsis* | Stems | Brassicaceae, oligophagous | Benedikt et al., 2010; Nikolai et al., 2018; Rheinheimer and Hassler, 2010 |
| *Ceutorhynchus constrictus* | Fruits | Brassicaceae, monophagous | Avgın and Colonnelli, 2011; Nikolai et al., 2018; Rheinheimer and Hassler, 2010 |
| *Ceutorhynchus nigritulus* | - | Brassicaceae, oligophagous | Rheinheimer and Hassler, 2010 |
| *Ceutorhynchus granulicollis* | Fruits | Brassicaceae, narrowly monophagous | Dieckmann, 1972; Rheinheimer and Hassler, 2010; Nikolai et al., 2018 |
| *Ceutorhynchus syrites* | Fruits | Brassicaceae, monophagous | Delbol, 2008; Delbol, 2013; Rheinheimer and Hassler, 2010; Nikolai et al., 2018 |
| *Ceutorhynchus squamulosus* |  | Brassicaceae, oligophagous | Sanz et l., 1996 |
| *Ceutorhynchus atomus* | Siliques, seeds and stem | Brassicaceae, oligophagous | Stelter, 1964; Delbol, 2008; Delbol, 2013; |
| *Ceutorhynchus querceti* | Fruits | Brassicaceae, monophagous | Anderson , 1997; Benedikt et al., 2010; Delbol, 2008; Delbol, 2013 |
| *Ceutorhynchus campestris* | Receptacle | Asteraceae, monophagous | Delbol, 2008; Delbol, 2013; Dieckmann, 1972; |
| *Ceutorhynchus triangulum* | Fflower and stem | Asteraceae, monophagous | Behne, 1987; Delbol, 2008; Delbol, 2013; Germann, 2005 |
| *Ceutorhynchus puncticollis* | stem | Brassicaceae, oligophagous | Behne, 1981; Dieckmann, 1972; Rheinheimer and Hassler, 2010; Nikolai et al., 2018 |
| *Ceutorhynchus tibialis* | - | Brassicaceae, monophagous | Benedikt et al., 2010; Sanz Benito et al., 1996; Compte, 1981 |
| *Ceutorhynchus sulcatus* | - | Brassicaceae, monophagous | Scheuch, 1930; Nikolai et al., 2018 |
| *Ceutorhynchus resedae* | Base of the stem | Resedaceae, monophagous | Colonnelli and Osella, 2009; Dieckmann, 1972; Delbol, 2008; Delbol, 2013 |
| *Ceutorhynchus dubius* | Root | Brassicaceae, monophagous | Behne, 1987; Dieckmann, 1972 |
| *Ceutorhynchus hutchinsiae* | - | Brassicaceae, monophagous | Benedikt et al., 2010; |
| *Ceutorhynchus scapularis* | Stem and root collar | Brassicaceae, oligophagous | Dieckmann, 1972; Rheinheimer and Hassler, 2010; Nikolai et al., 2018 |
| *Ceutorhynchus leprieuri* | Leaf disk | Brassicaceae, oligophagous | Benedikt et al., 2010; Dieckmann, 1972 |
| *Ceutorhynchus pectoralis* | Stem | Brassicaceae, monophagous | Buhr, 1964; Dieckmann, 1972; |
| *Ceutorhynchus coerulescens* | Stem and petiole | Brassicaceae, monophagous | Benedikt, 2014; Delbol, 2008; Delbol, 2013; Dieckmann, 1972 |
| *Ceutorhynchus thomsoni* | - | Brassicaceae, oligophagous | Dieckmann, 1972 |
| *Ceutorhynchus wellschmiedi* | - | Brassicaceae, monophagous | Hinz and Diaconu, 2015 |
| *Ceutorhynchus erysimi* | Petiole, leaf and stem | Brassicaceae, oligophagous | Compte 1981; Delbol, 2008; Delbol, 2013; Avgın and Colonnelli, 2011 |
| *Ceutorhynchus punctiger* | Flower and fruits | Asteraceae, monophagous | Anderson, 1997; Dieckmann, 1972; Dieckmann and Herger, 1985 |
| *Ceutorhynchus fennicus* | Flower | Asteraceae, oligofaag | Dieckmann, 1972; Benedikt et al., 2010 |
| *Ceutorhynchus assimilis* | Siliques and seeds | - | McLeo, 1953 |

1. Abbazzi P, Bartolozzi L, Crudele G, Sforzi A, 2003. I Coleotteri Curculionoidea del parco nazionale delle Foreste Casentinesi, Monte Falteronae Campigna (Insecta Coleoptera): 1° contributo. Redia 86: 81-95.
2. Anderson RS & Korotyaev BA, 2004a. Some Palearctic weevils in the subfamily Ceutorhynchinae (Coleoptera, Curculionidae) recently discovered in North America. – The Canadian Entomologist 136: 233-239.
3. Anderson RS, 1997. Weevils (Coleoptera: Curculionoidea, excluding Scolytinae and Platypodinae) of the Yukon. In: Insects of the Yukon. Biological Survey of Canada (Terrestrial Arthropods) (Danks HV & Downes JA, eds): 523–562.
4. Avgın SS & Colonnelli E, 2011. Curculionoidea (Coleoptera) from southern Turkey-African Journal of Biotechnology 10(62): 13555-13597.
5. Aydın E & Hacet N, 2016. On some Ceutorhynchinae (Coleoptera: Curculionidae) from Turkish Thrace region, with new records to Turkish fauna. - Journal of the entomological Research Society 18(2): 85-94.
6. Balalaikins M, 2012. Curculionidae (except Scolytinae and Platypodinae) in Latvian fauna, taxonomical structure, biogeography and forecasted species. – Acta biologica Universitatis daugavpiliensis 12(4): 67–83.
7. Behne L, 1981. Zur Verbreitung und Biologie bemerkenswerter Rüsselkäfer im Bezirk Magdeburg. – Entomologische Nachrichten 25(7/8): 113-120.
8. Behne L, 1987. Beitrag zur Kenntnis der Rüsselkäferfauna des NSG “Silberberge” bei Gartz/Oder (Col., Curculionidae). – Entomologische Nachrichten und Berichte 31(1): 25-28.
9. Benedikt S, 2014. [Contribution to the knowledge of beetles (Coleoptera) from the Jelšavský kras (karst) (Slovakia)]. – Západočeské entomologické Listy 5: 32–90.
10. Benedikt S, Borovec R, Fremuth J, Krátký J, Schön K, Skuhrovec J & Trýzna M, 2010. Annotated checklist of weevils (Coleoptera: Curculionoidea excepting Scolytinae and Platypodinae) of the Czech Republic and Slovakia. 1. Systematics, faunistics, history of research on weevils in the Czech Republic and Slovakia, structure outline, checklist. Comments on Anthribidae, Rhynchitidae, Attelabidae, Nanophyidae, Brachyceridae, Dryophthoridae, Erirhinidae and Curculionidae: Curculioninae, Bagoinae, Baridinae, Ceutorhynchinae, Conoderinae, Hyperinae. – Klapalekiana 46: 1-363.
11. Berg C van den & Sande JC van de, 1999. Ceutorhynchus turbatus new for The Netherlands (Coleoptera: Curculionidae). – Entomologische Berichten 59(10): 157-159.
12. Buhr, H 1964. Bestimmungstabellen der Gallen (Zoo-und Phytocecidien) a Pflanzen Mittel- und Nordeuropas. 1. A-M. Fischer, Jena.
13. Colonnelli E & Osella G, 2009. New data on some Curculionoidea (Coleoptera: Anthribidae, Apionidae, Curculionidae) from Sardinia. – Zootaxa 2318: 421-426.
14. Colonnelli E, 1990. Curculionidae Ceutorrhynchinae from the Canaries and Macaronesia (Coleoptera). – Vieraea 18: 317-337.
15. Compte A, 1981. Coleópteros del monte de El Pardo y la Casa de Campo (Madrid). 1. Superfamilia Curculionoidea. Eos 57: 17-67.
16. Delbol M, 2008. Liste partielle des Ceutorhynchini (Curculionidae: Ceutorhynchinae) de Belgique. Faunistic Entomology, Entomologie faunistique 61(3): 109-123.
17. Delbol M, 2013. Catalogue des Curculionoidea de Belgique (Coleoptera: Polyphaga). – Belgian Journal of Entomology 13: 1-95.
18. Dieckmann L and Herger P, 1985. Zur Insektenfauna der Umgebung der Vogelwarte Sempach, Kanton Luzern, 16. Coleoptera 4: Curculionidae. Entomologische Berichte Luzern 13: 81-85. Zur Insektenfauna der Umgebung der Vogelwarte Sempach, Kanton Luzern, 16. Coleoptera 4: Curculionidae. Entomologische Berichte Luzern 13: 81-85
19. Dieckmann L, 1972. Beiträge zur Insektenfauna der DDR: Coleoptera: Curculionidae: Ceutorhynchinae. – Beiträge zur Entomologie 22(1/2): 3-128.
20. Gebiola M, Bernardo U, Ribes A & Gibson GAP, 2015. An integrative study of Necremnus Thomson (Hymenoptera: Eulophidae) associated with invasive pests in Europe and North America: taxonomic and ecological implications. – Zoological Journal of the linnean Society 173: 352–423.
21. Germann Ch, 2005. Beitrag zur Gattung Microplontus Wagner, 1944 in der Schweiz: mit der Meldung zweier weiterer Arten (Coleoptera, Curculionidae, Ceutorhynchinae). – Mitteilungen der schweizerischen entomologischen Gesellschaft 78(3/4): 325-332.
22. Germann Ch, 2011. Supplement zur Checkliste der Rüsselkäfer der Schweiz (Coleoptera, Curculionoidea). – Mitteilungen der schweizerischen entomologischen Gesellschaft 84: 155–169.
23. Gültekin L. 2014. Spring fauna of Ceutorhynchinae (Coleoptera: Curculianidae) weevils from Southeast Mediterranean Turkey. Journal of the Entomological Research Society 16(1): 49-59.
24. Heijerman Th, 1993. Naamlijst van de snuitkevers van Nederland en het omliggend gebied (Curculionoidea: Curculionidae, Apionidae, Attelabidae, Urodontidae, Anthribidae en Nemonychidae). – Nederlandse faunistische Mededelingen 5: 19-46.
25. Hinz HL & Diaconu A, 2015a. Biology and ﬁeld host range of Ceutorhynchus cardariae, a potential biological control agent for Lepidium draba. – Journal of applied Entomology 139: 168-178.
26. Laffin, Richard D., 2005. Population structure and variation in Ceutorhynchus obstrictus and C. neglectus (Coleoptera: Curculionidae)  University of Alberta (Canada) ProQuest Dissertations Publishing. MR09211.
27. Mason, P., Miall, J., Bouchard, P., Brauner, A., Gillespie, D., & Gibson, G. 2014. The parasitoid communities associated with Ceutorhynchus species (Coleoptera: Curculionidae) in Ontario and Québec, Canada. The Canadian Entomologist, 146(2), 224-235. doi:10.4039/tce.2013.65
28. McLeod, J.H. 1953. Notes on the cabbage seedpod weevil, Ceutorhynchus assimilis (Payk.) (Coleoptera: Curculionidae), and its parasites. Proceedings of the Entomological Society of British Columbia, 49: 11–18.
29. New data on two similar species of Cionus Clairville & Schellenberg, 1798 (Coleoptera, Curculionidae) from Lithuania. – Acta zoologica lituanica 20(4): 225-228.
30. Nikolai Yunakov and Vitalij Nazarenko and R. V. Filimonov and Semyon V. Volovnik. 2018. A survey of the weevils of Ukraine (Coleoptera: Curculionoidea). Zootaxa, 2018,4404(1),1-494
31. Nilsson G, 2005. Zur Bionomie von Ceutorhynchus sophiae (Stephehs, 1829) (Col., Curculionidae) Entom ologische Nachrichten und Berichte 49(2): 133-136.
32. Reibnitz J, 2012. Neu- und Zusatzmeldungen zum Verzeichnis der Käfer Baden-Württembergs 1950–2000. – Mitteilungen des entomologischen Vereins Stuttgart 47: 23-39.
33. Rheinheimer, J. and M.Hassler.2010. DieRüsselkäferBaden-Württembergs. Verlag Regionalkultur.
34. Sakine Serap Avgın and Enzo Colonnelli, Curculionoidea (Coleoptera) from southern Turkey.African Journal of Biotechnology Vol. 10(62), pp. 13555-13597, 12 October, 2011
35. Sanz Benito MJ, García-Ocejo Izquierdo, A & Mozos Pascual M de los, 1996a. Contribución al conocimiento faunístico y biológico de los curculiónidos (Coleóptera, Curculionoidea) de la provincia de Madrid. Boletín de la Asociación española de Entomología 20(1-2): 119-139.
36. Scheuch H, 1930. Mitteilungen über Nährpflanzen einheimischer Ceuthorrhynchinen. Koleopterologische Rundschau 16(5): 172-176.
37. Stelter H & Buhr H, 1964. Untersuchungen über Gallmücken. 12. Rhabdophaga clavifex (Kieffer, 1892), Rhabdophaga repenticola Stelter n. sp. und ihre Gallen (Diptera: Itonidae/Cecidomyiidae). – Beiträge zur Entomologie 14: 71-84.


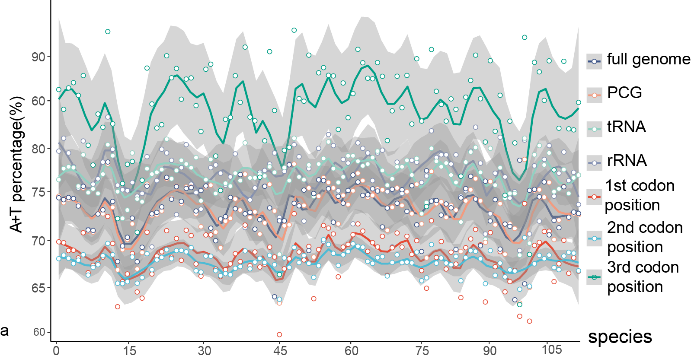

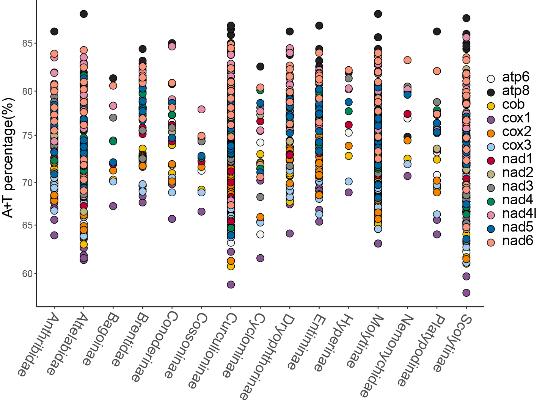


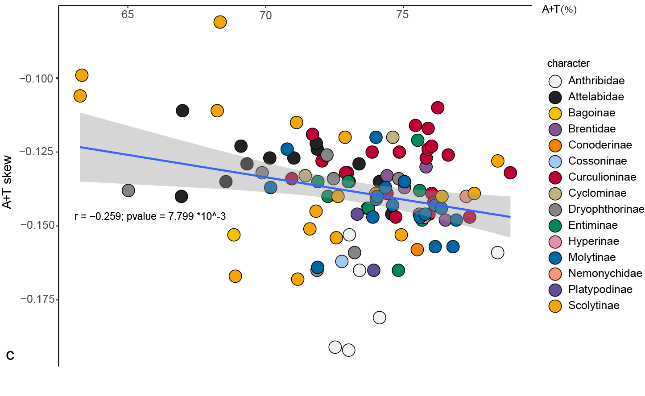

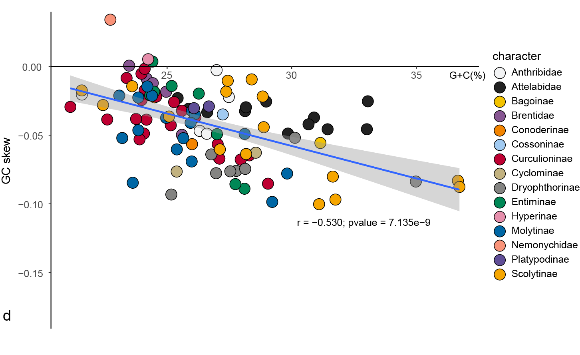


**Fig. S1.** Analyses of nucleotide compositions for Curculionoidea species. (a) Comparison of A+T contents in components of mitochondrial genomes for all species analyzed. (b) Comparison of A+T contents for 13 protein-coding genes (PCGs). (c) Correlations between (A+T)% and AT-skew in 13 PCGs. (d) Correlations between (G+C)% and GC-skew in 13 PCGs.


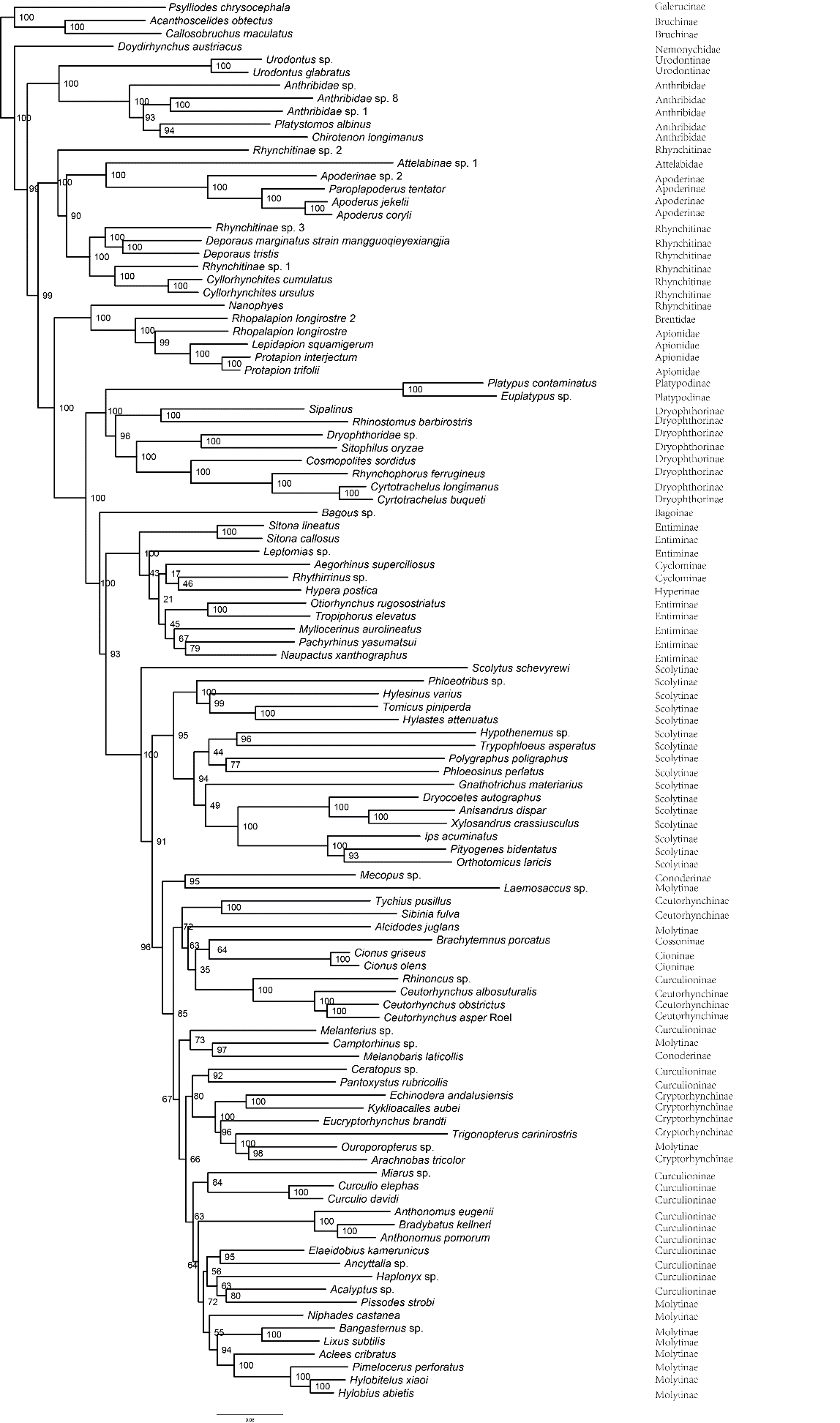


**Fig. S2.** The phylogenetic tree inferred from the PCG12 matrix based on the partitioning model using IQ-TREE


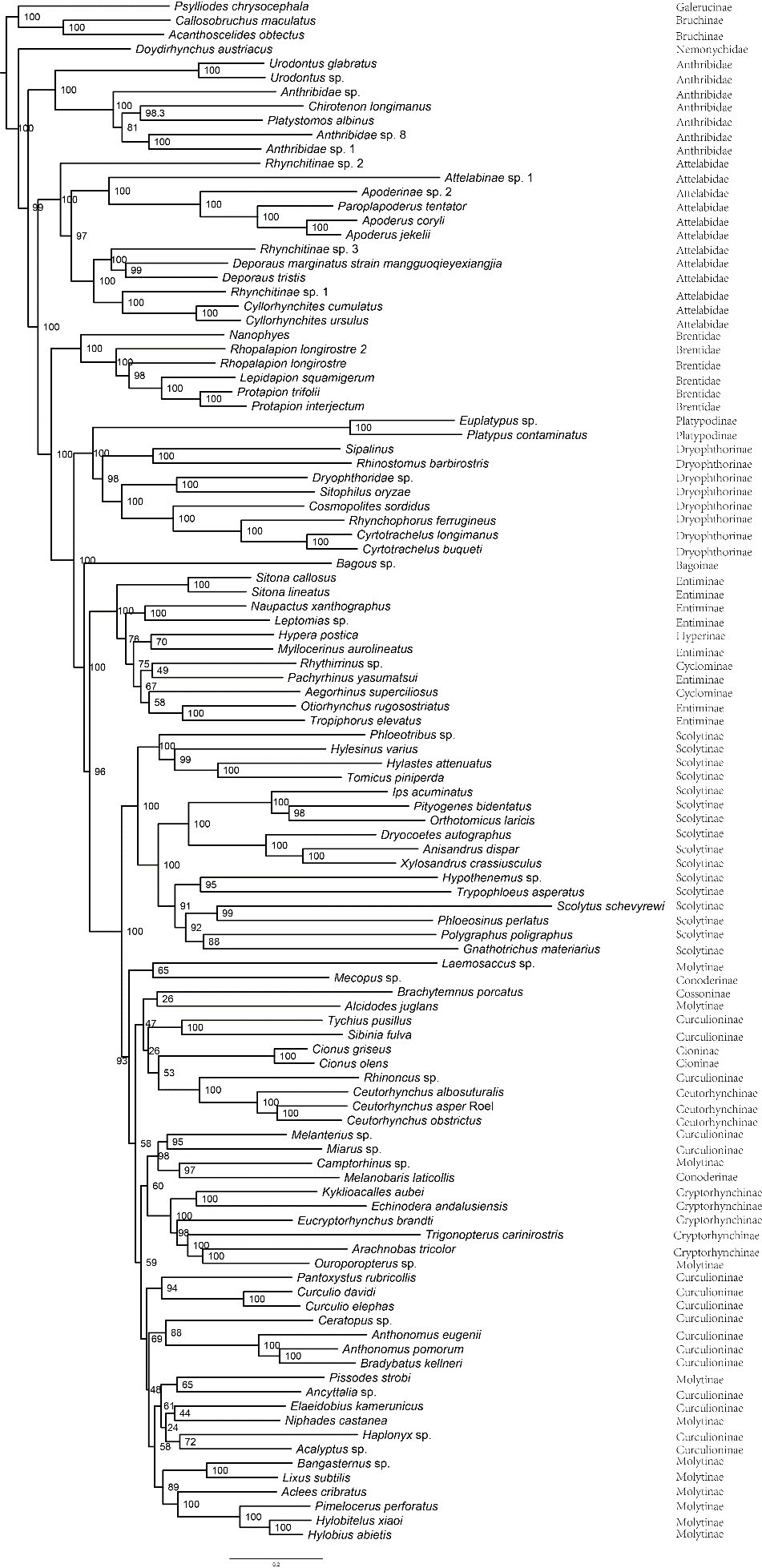


**Fig. S3**. The phylogenetic trees inferred from the PCG matrix based on the partitioning model using IQ-TREE


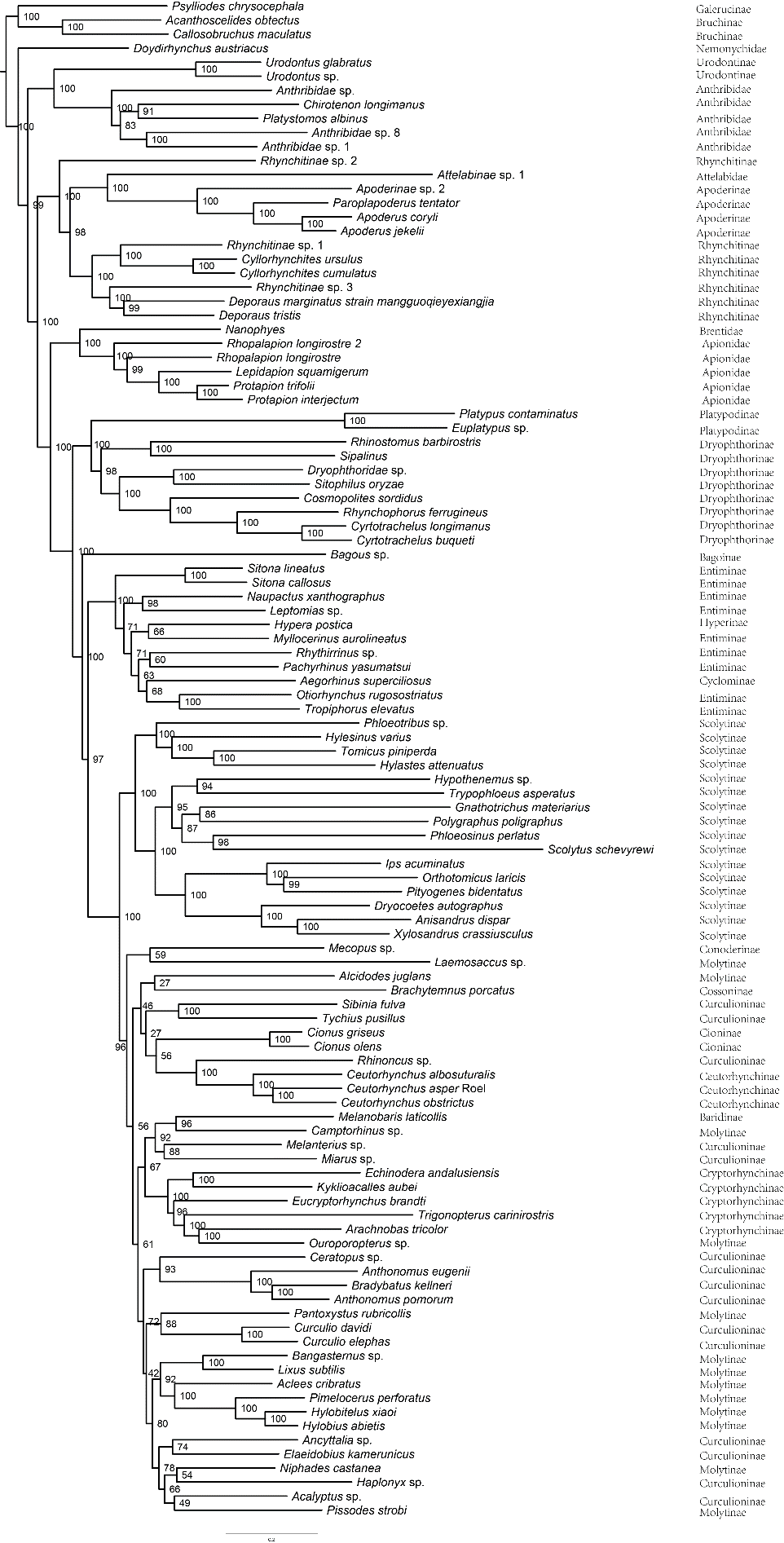


**Fig. S4**. Phylogenetic tree inferred from the ALL matrix based on the partitioning model using IQ-TREE


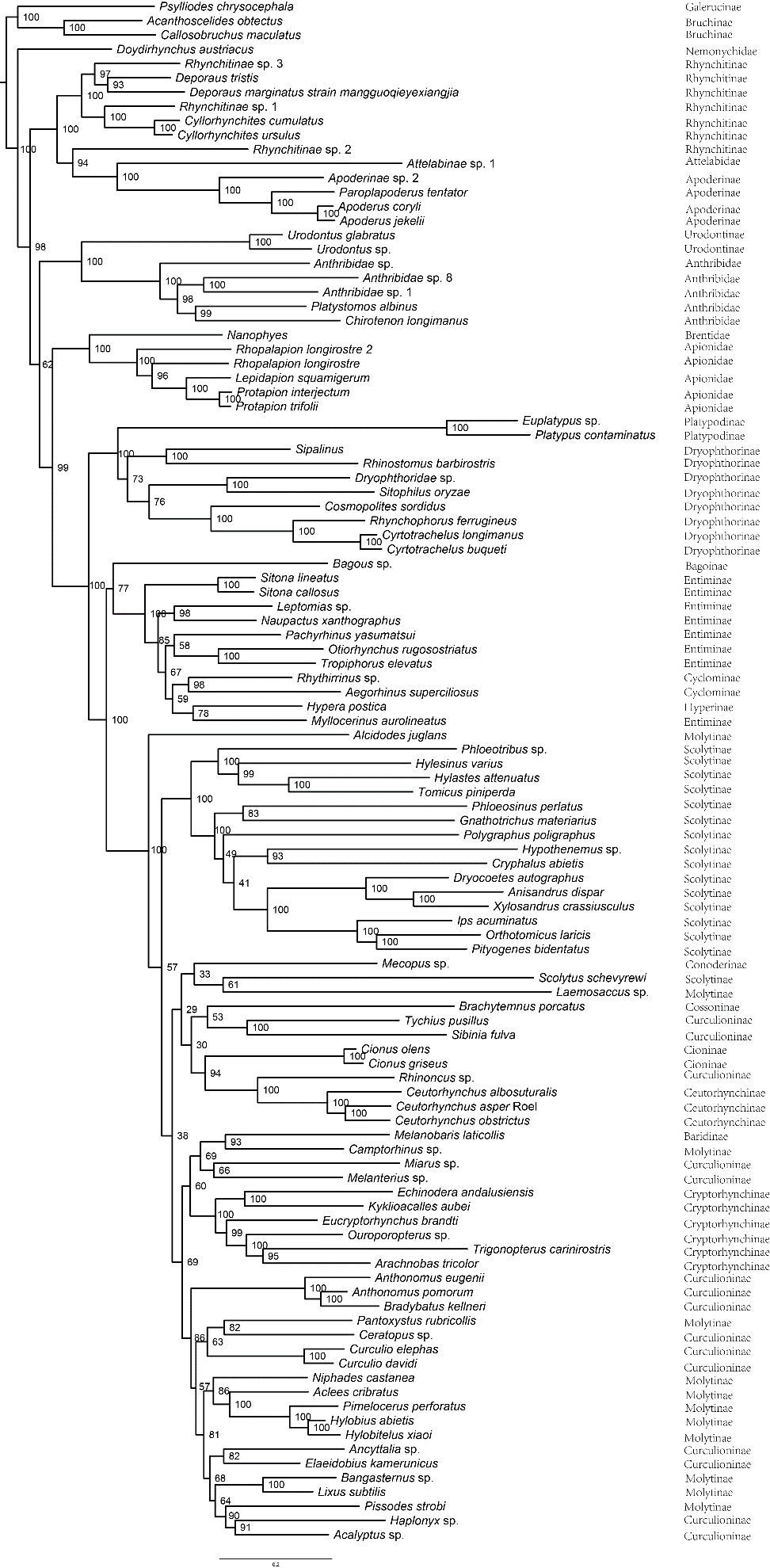


**Fig. S5**. Phylogenetic tree inferred from the PCG matrix based on the site-heterogeneous model (LG) using IQ-TREE


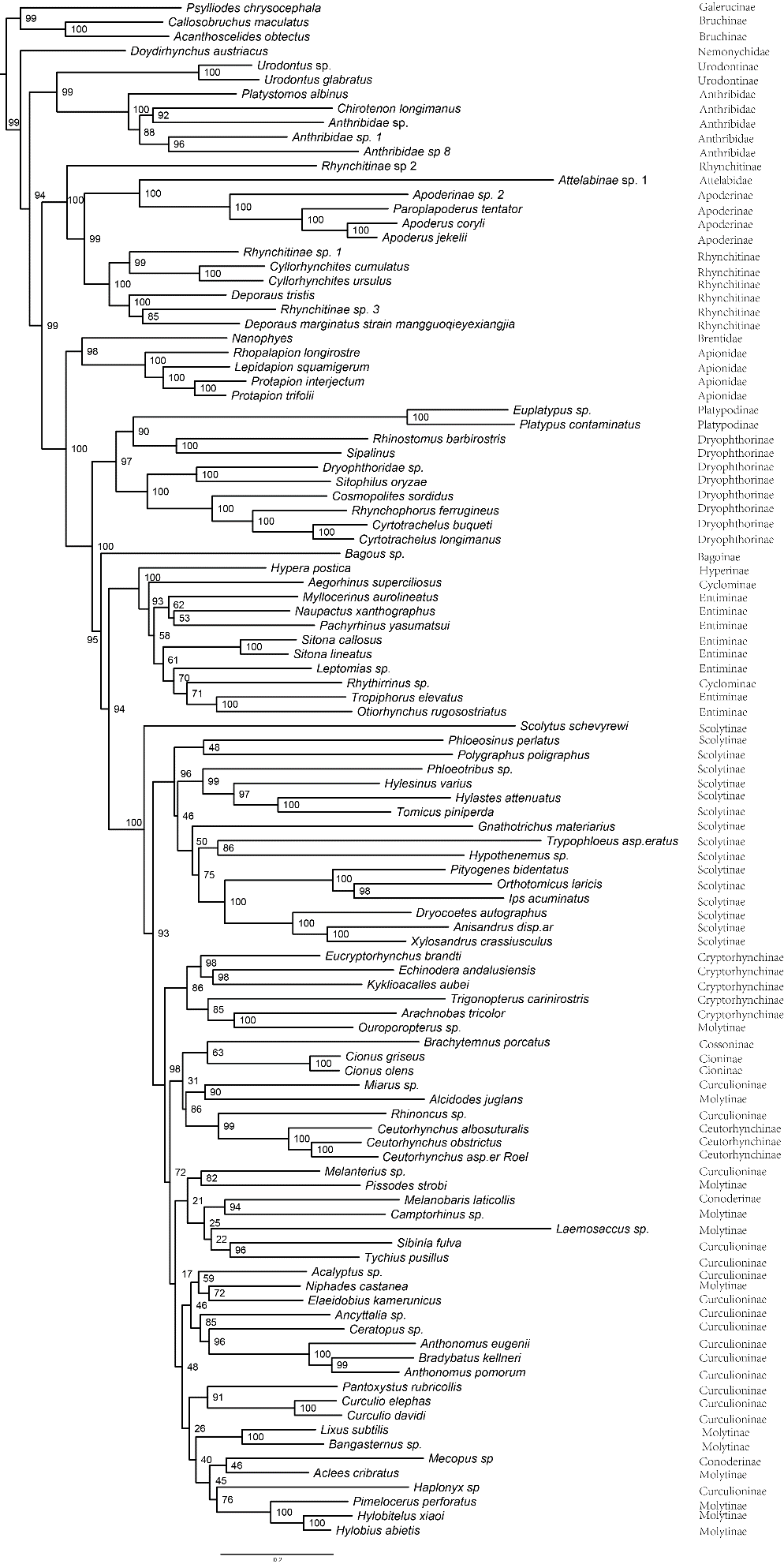


**Fig. S6**. Phylogenetic tree inferred from the SRH matrix based on the partitioning model using IQ-TREE


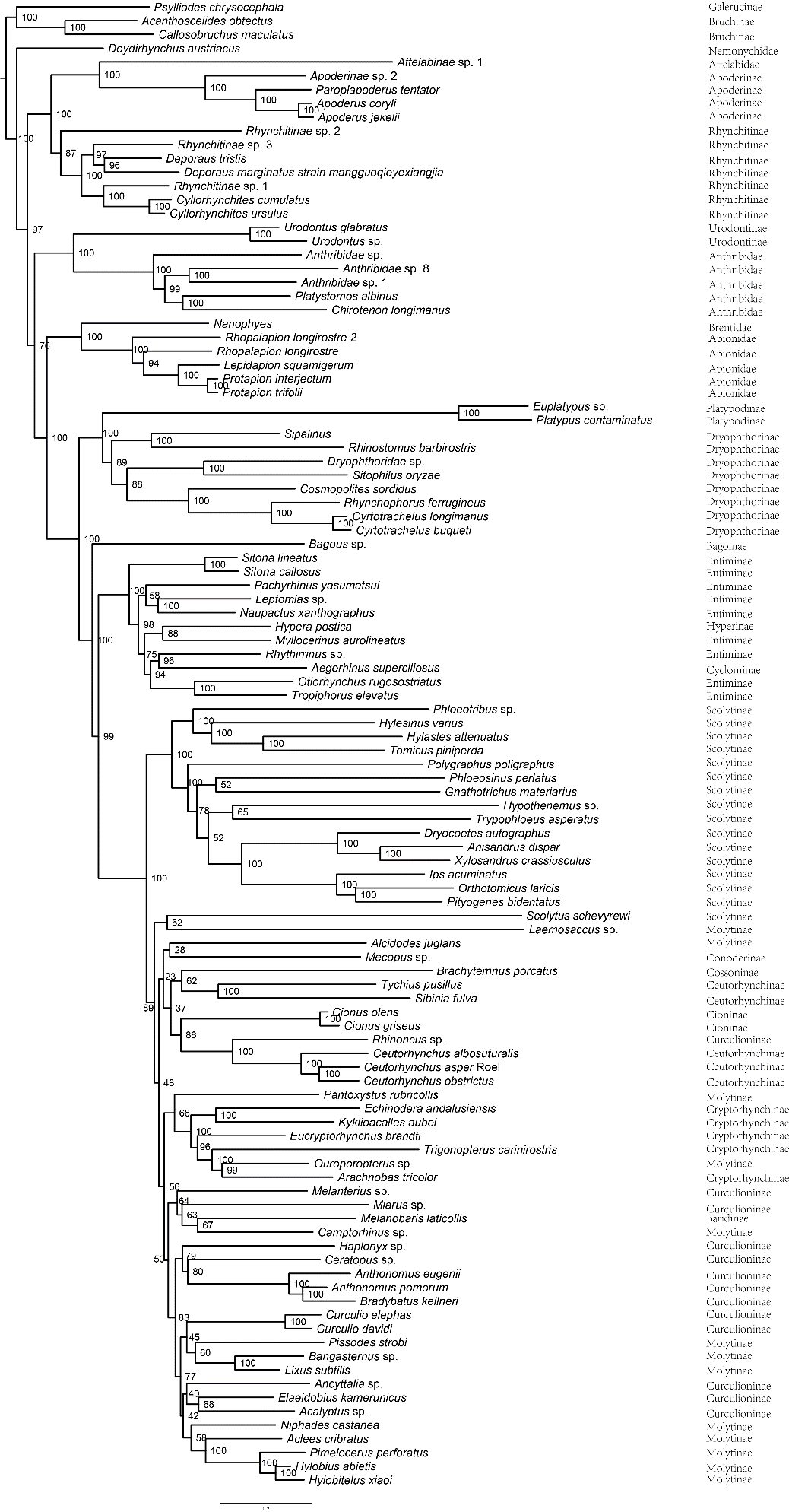


Fig. S7. Phylogenetic tree inferred from the PCG matrix based on the site-heterogeneous model (LG + C60) using IQ-TREE


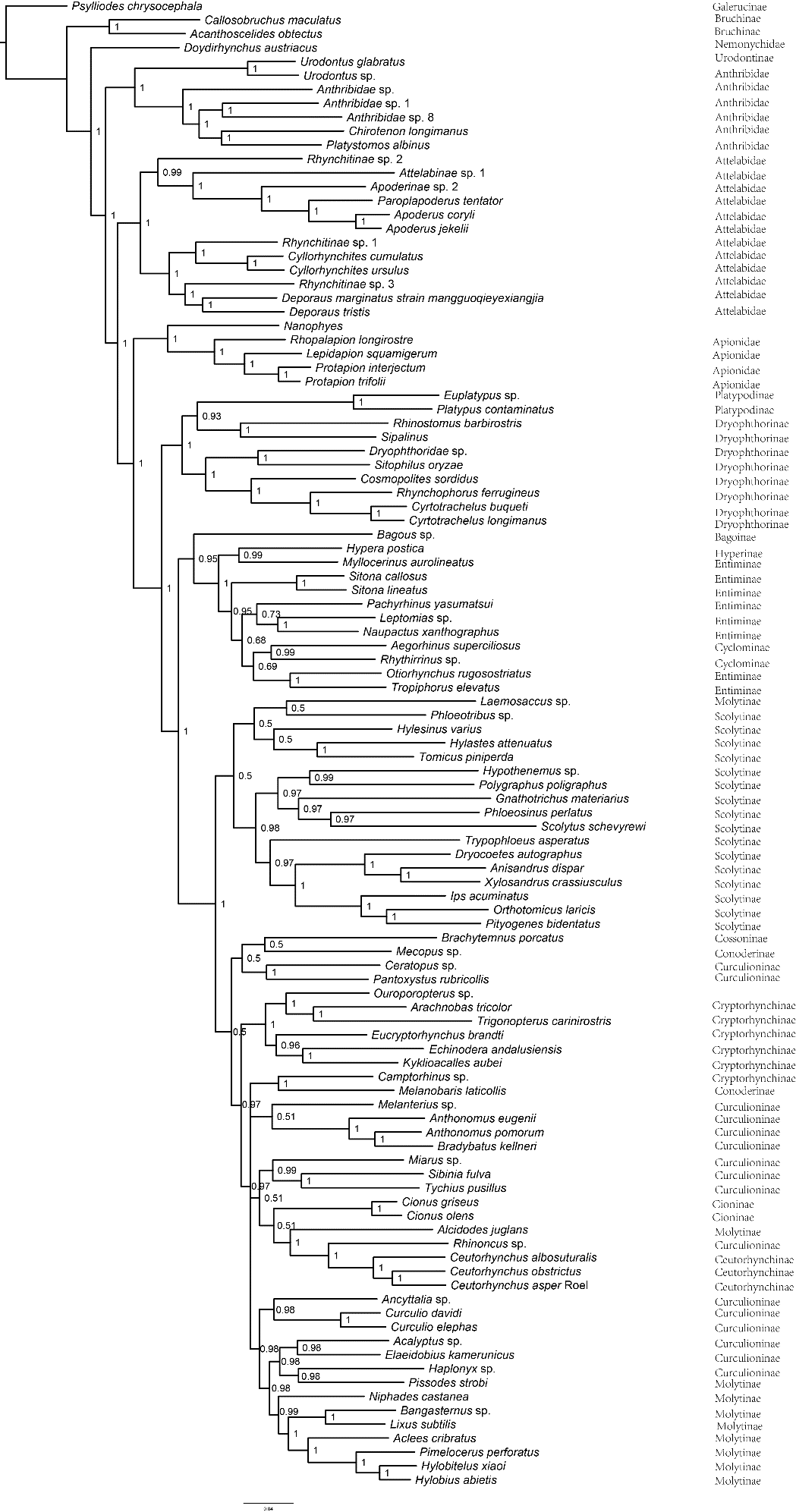


**Fig. S8**. Phylogenetic tree inferred from the PCG12 matrix using MrBayes


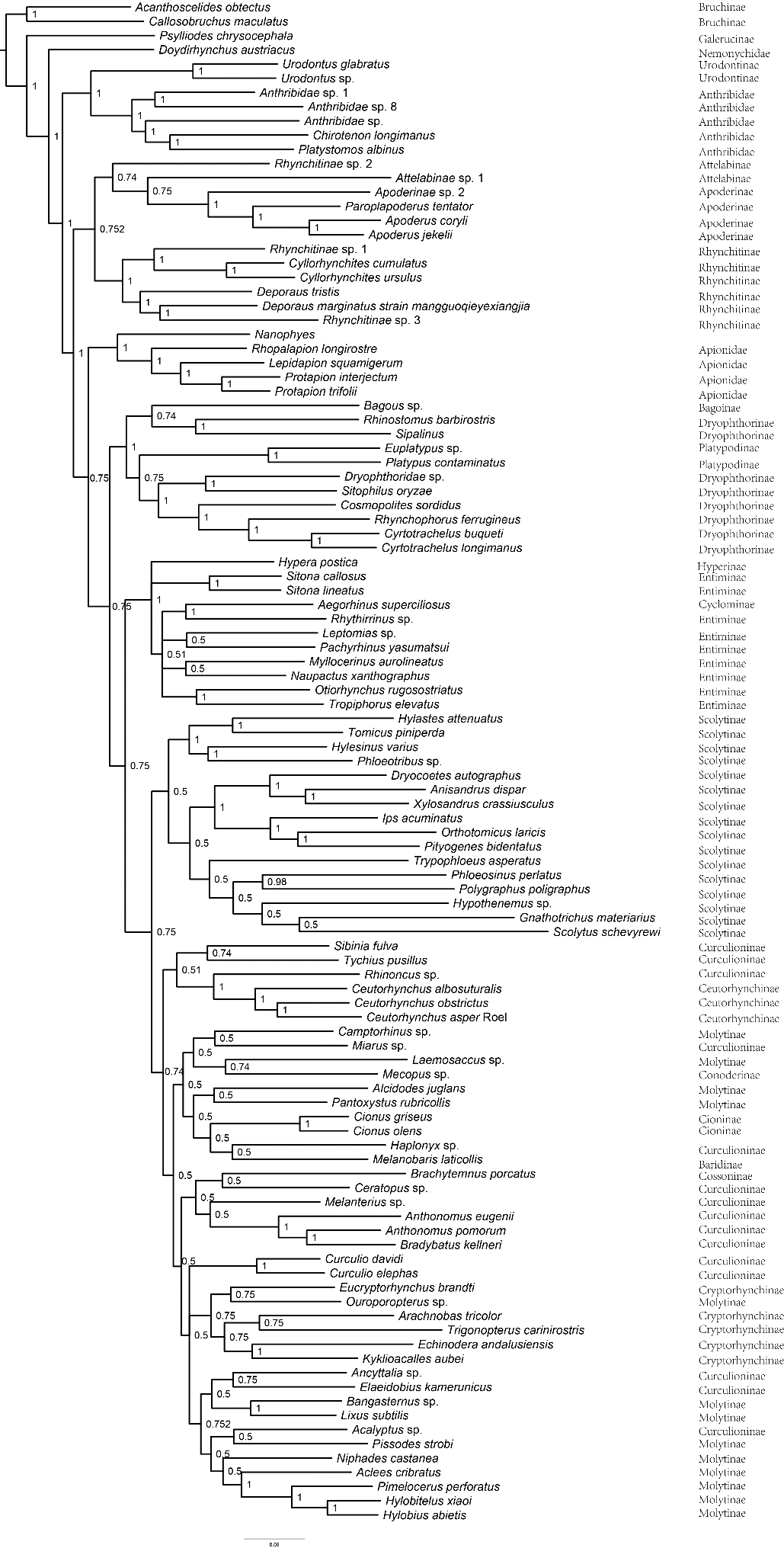


**Fig. S9**. Phylogenetic tree inferred from the PCG matrix using MrBayes


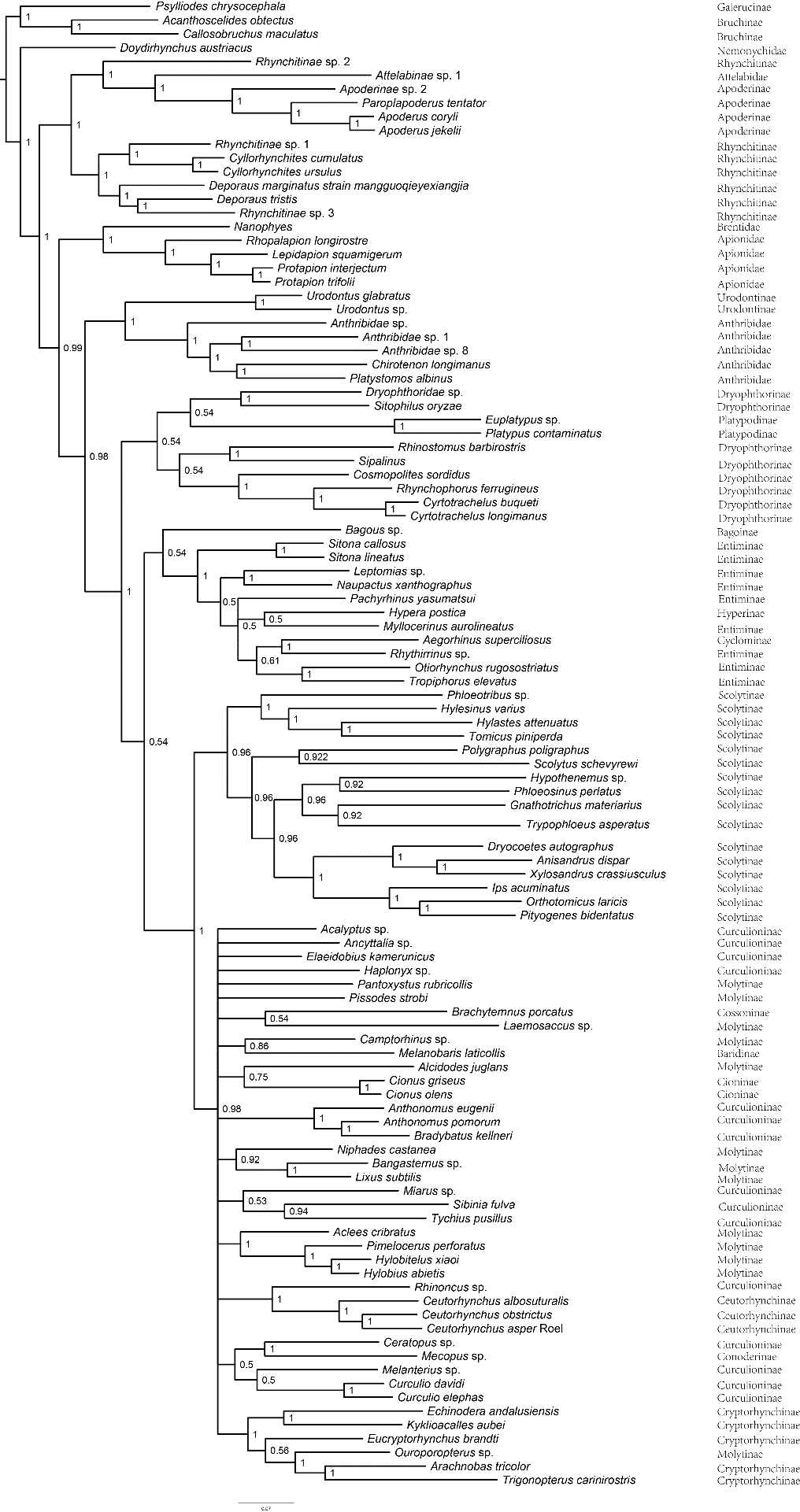


**Fig. S10**. Phylogenetic tree inferred from the PCGAA matrix using MrBayes


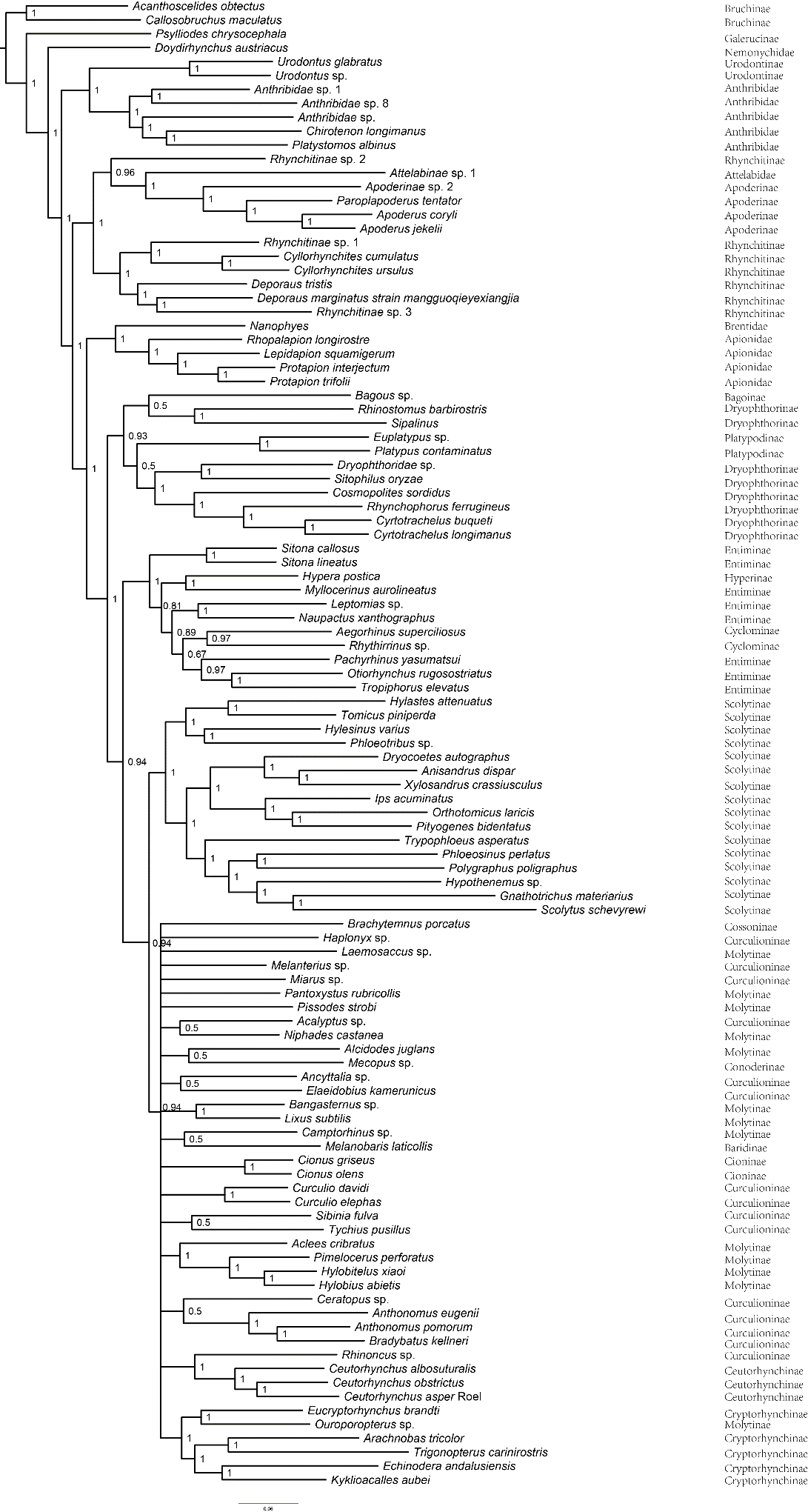


**Fig. S11**. Phylogenetic tree inferred from the SRH matrix using MrBayes


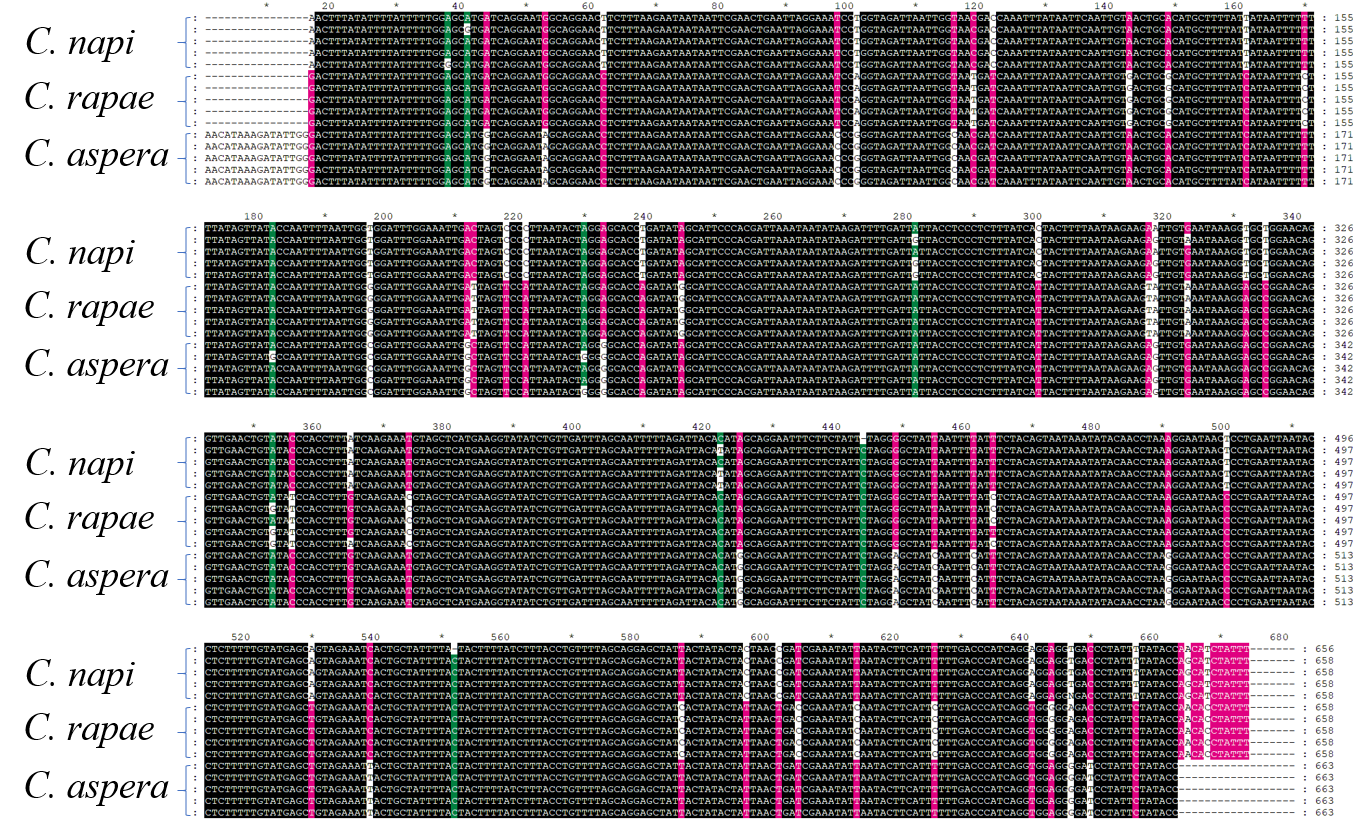


**Fig. S12**. Alignment of representative *cox1* sequences of three *Ceutorhynchus* species (*C. napi*, *C. rapae*, and *C. asper*). Black areas represent conserved areas, green areas represent sequence identity of 75-99%, and red areas represent sequence identity of 50-74%.
